# Supplementary material for: Bright probes, blurred metabolism: Navigating fluorescent protein cross-excitation in NADH FLIM
Source: Biophys Rep (N Y). 2026 Oct 20;6(4):100294. doi: 10.1016/j.bpr.2026.100294 (PMC13769086; doi:10.1016/j.bpr.2026.100294)
Supplement: Supplementary file 1 [file mmc1.pdf]

Biophysical Reports, Volume *6*

Supplemental information

Bright probes, blurred metabolism: Navigating fluorescent protein cross-excitation in NADH FLIM

Janielle Cuala, Oscar Alberto de la Fuente, Lynne Cherchia, Yuanzhong Pan, Tvisha Singh, Chris Deng, Andrea Velazquez, Kate White, Senta K. Georgia, Steve A. Kay, Scott E. Fraser, and Falk Schneider

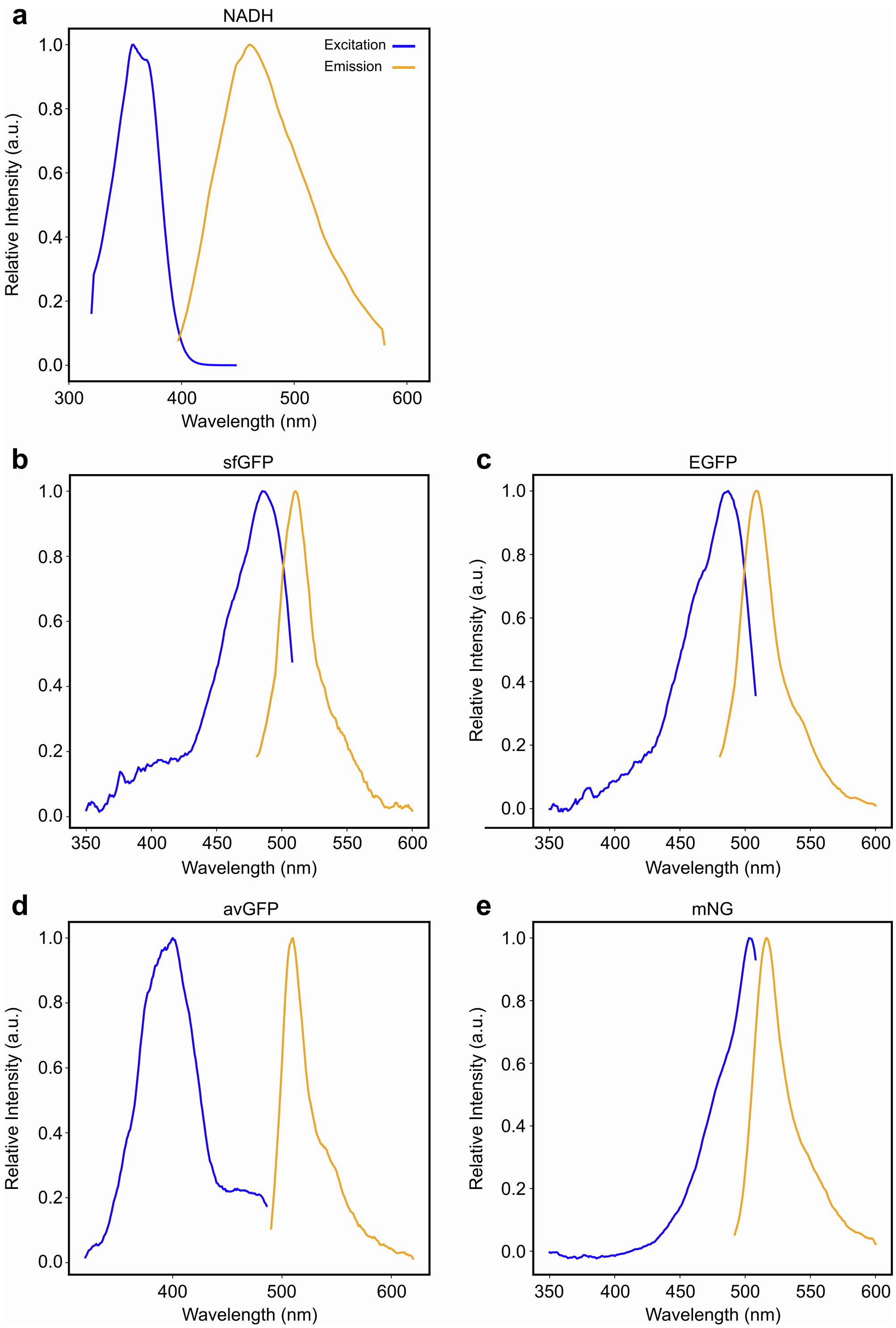

Figure S1: One photon excitation and emission spectra of NADH in solution and green fluorescent proteins expressed in U2OS cells as H2B fusions. Blue curves are the excitation spectra, and the yellow curves are the emission spectra.
a NADH in water
b sfGFP in cells
c mEGFP

d avGFP
e mNG
Spectra were acquired in glass bottom 96 well plates on a fluorescence plate reader. Lines represent averages of multiple repeats.

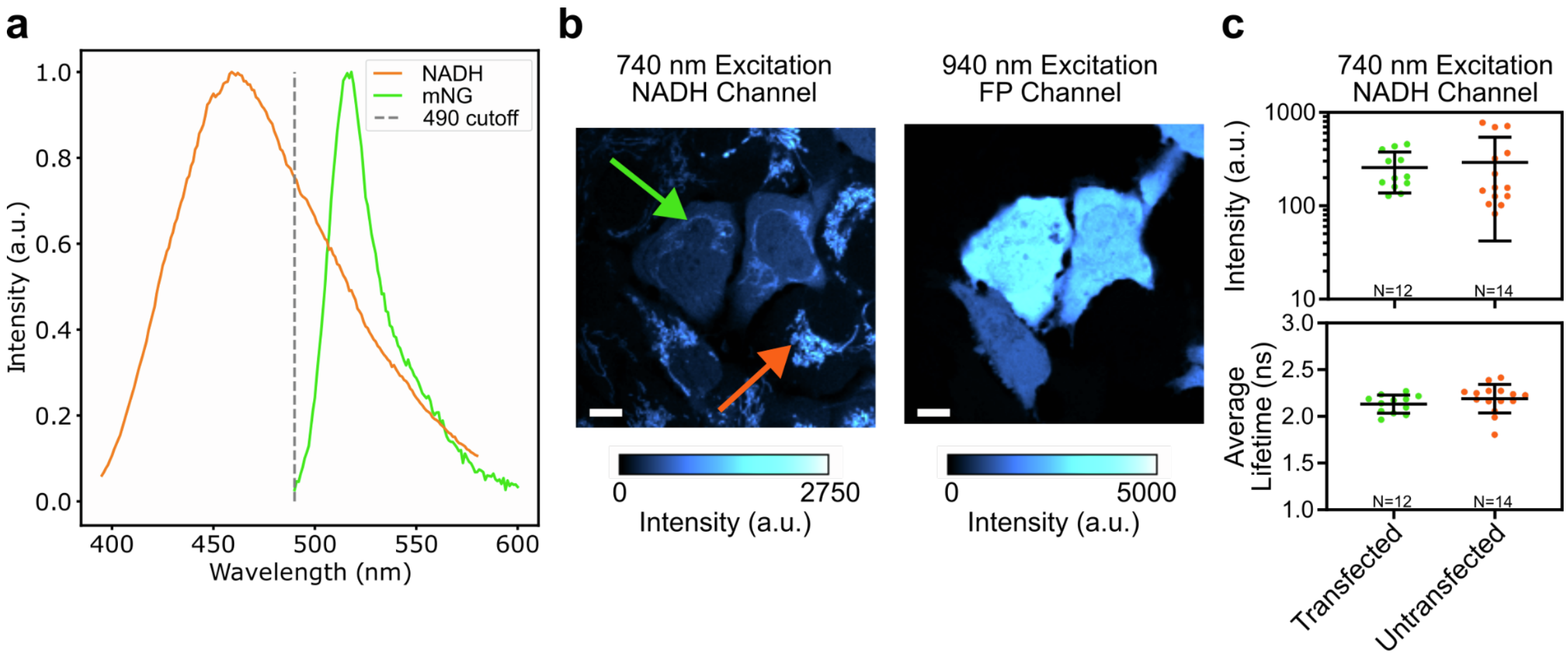

Figure S2: Cross-talk of NADH and mNeonGreen (mNG).

a Spectral overlap of NADH (in water) and mNG-H2B expressed in HEK cells

b Laser scanning TPE microscopy images of cells expressing cytoplasmic mNG. Left: Excitation with 740 nm. Right: Excitation with 940 nm. Arrows indicate mitochondria in transfected (green) and untransfected (orange) cells. Scale bars are 10 µm.

c Fluorescence intensity and lifetime of segmented cytoplasm in transfected (green) and untransfected (orange) cells. Every dot represents one cell. Horizontal bar is mean value and vertical bars standard deviation.

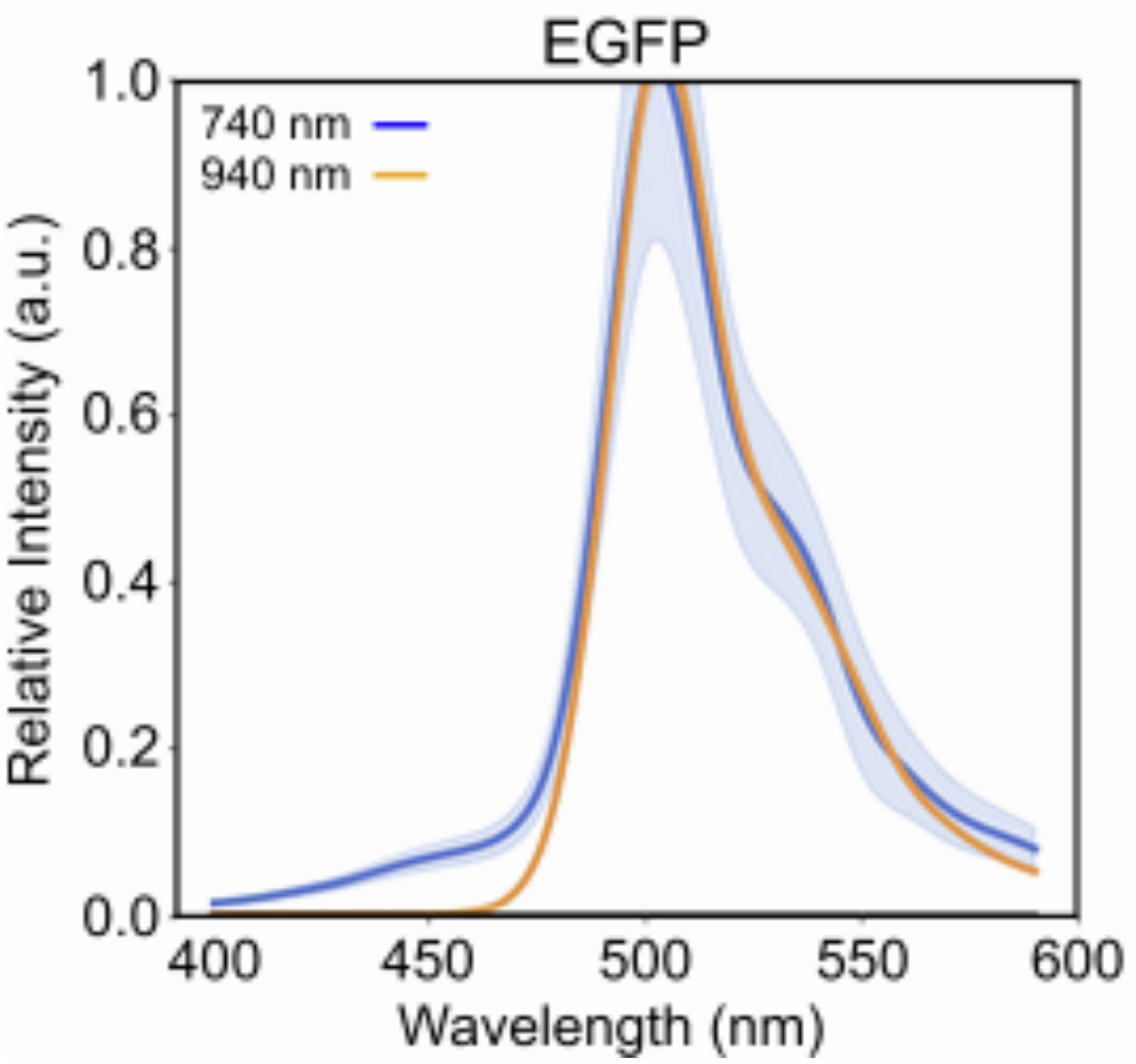

Figure S3: Two-photon emission spectra of recombinant EGFP in L-15.
150 nM solution of EGFP in L-15 was excited using 740 nm (blue) and 940 nm (orange). Values are averaged across an image and the shaded area represents standard deviation.

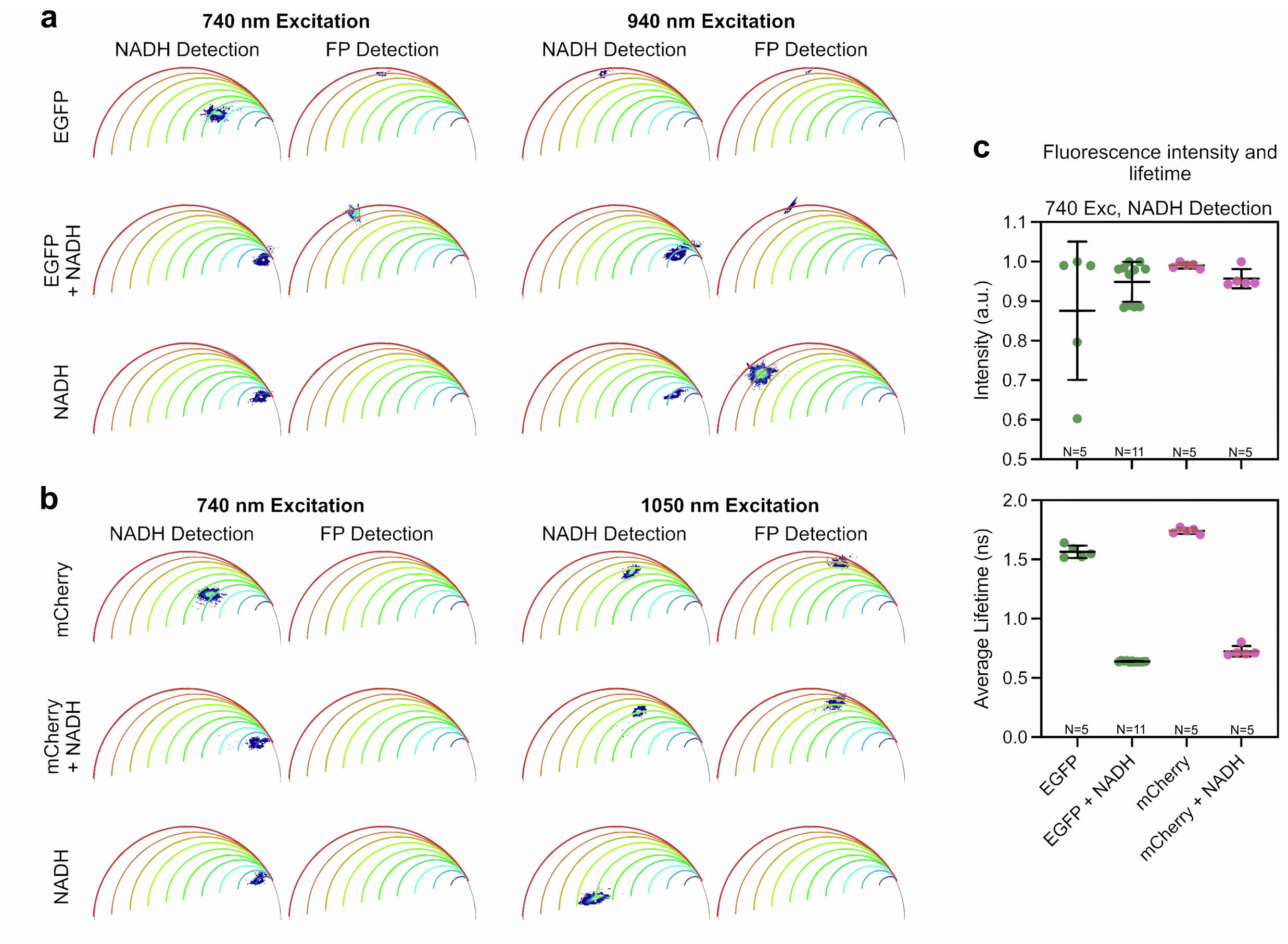

Figure S4: Excitation of purified recombinant EGFP and mCherry in the presence and absence of NADH in L-15. EGFP is imaged at a concentration of 150 nM, mCherry is imaged at 250 nM, and NADH is imaged at 200 µM.

a Phasors for recombinant EGFP and NADH fluorescence lifetime. Left: Excitation with 740 nm and NADH detection (410-490 nm, left) and FP detection (500-600 nm, right). Right: Excitation with 940 nm and NADH detection (left) and FP detection (right).

b Phasors for recombinant mCherry and NADH fluorescence lifetime. Left: Excitation with 740 nm and NADH detection (410-490 nm, left) and FP detection (600-700 nm, right). Right: Excitation with 1050 nm and NADH detection (left) and FP detection (right).

c Fluorescence lifetime and intensity of recombinant FPs with 740 nm excitation and NADH detection. Horizontal bars indicate mean value and vertical bars indicate standard deviation.

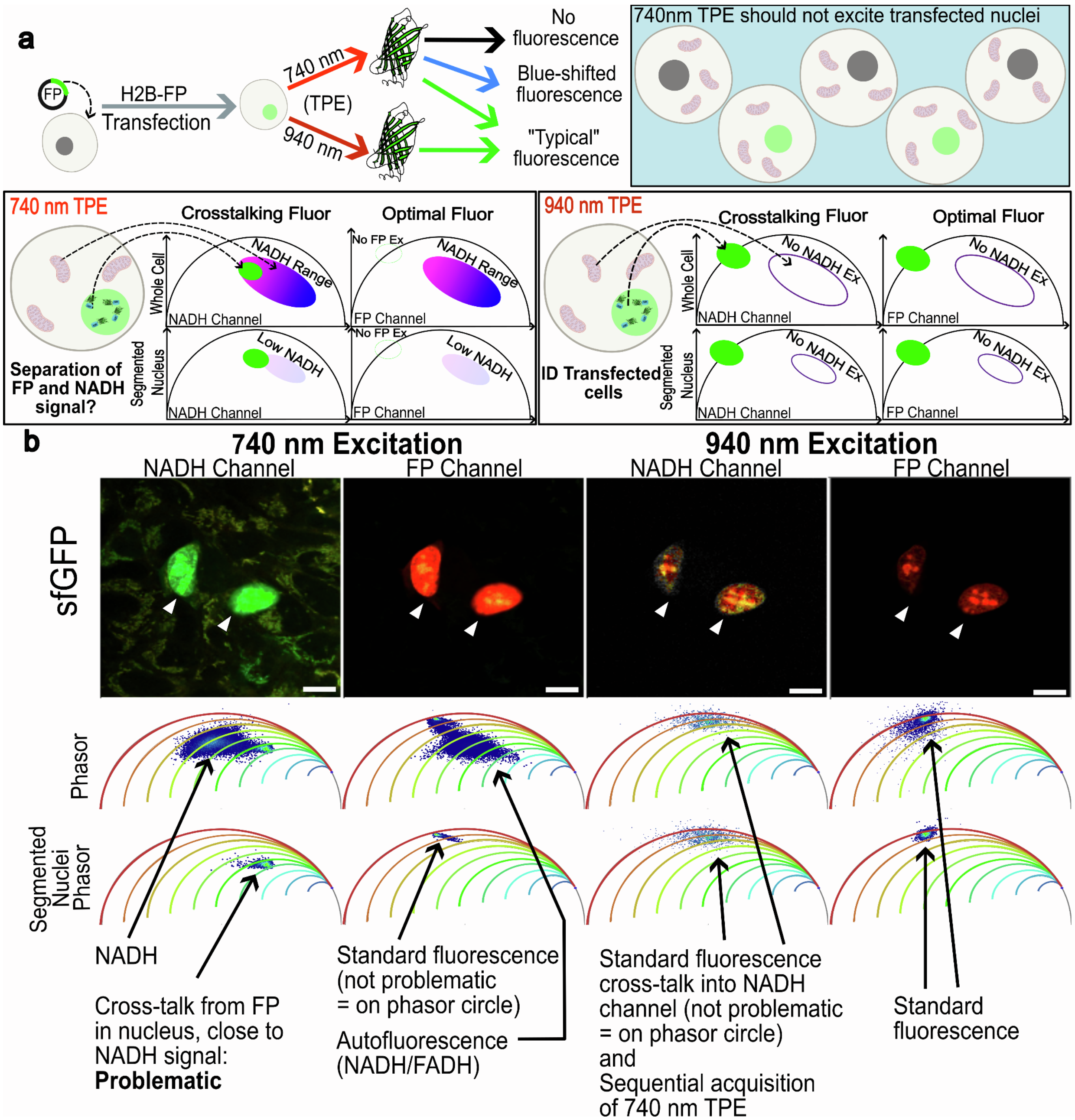

Figure S5: Cross-excitation assay.

a Extended cartoon explaining the cross-excitation assay (see also Fig. 2a).

b Cross-excitation assay of sfGFP same as in Fig. 2b,c with added annotations to aid data interpretation.

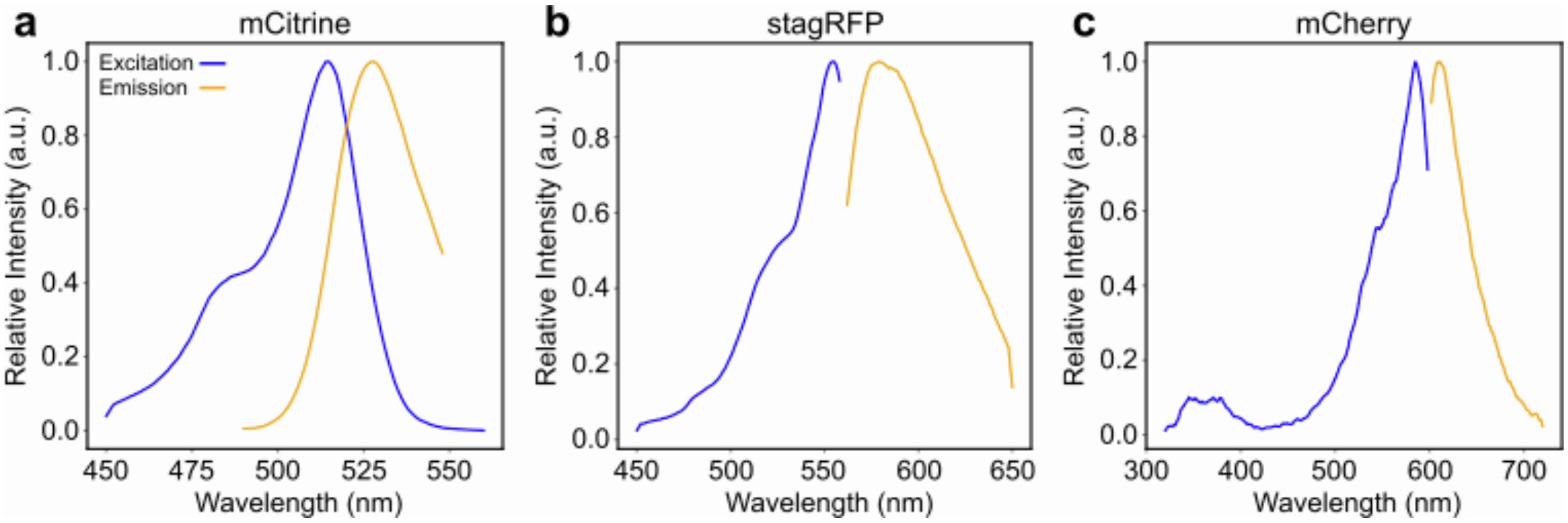

Figure S6: One photon excitation spectra of yellow and red-shifted FPs
Fluorescent proteins expressed in U2OS cells as H2B fusions. Blue curves are the excitation spectra, and the yellow curves are the emission spectra.

a mCitrine

b stagRFP

c mCherry

Spectra were acquired in glass bottom 96 well plates on a fluorescence plate reader. Curves represent averages of multiple repeats.

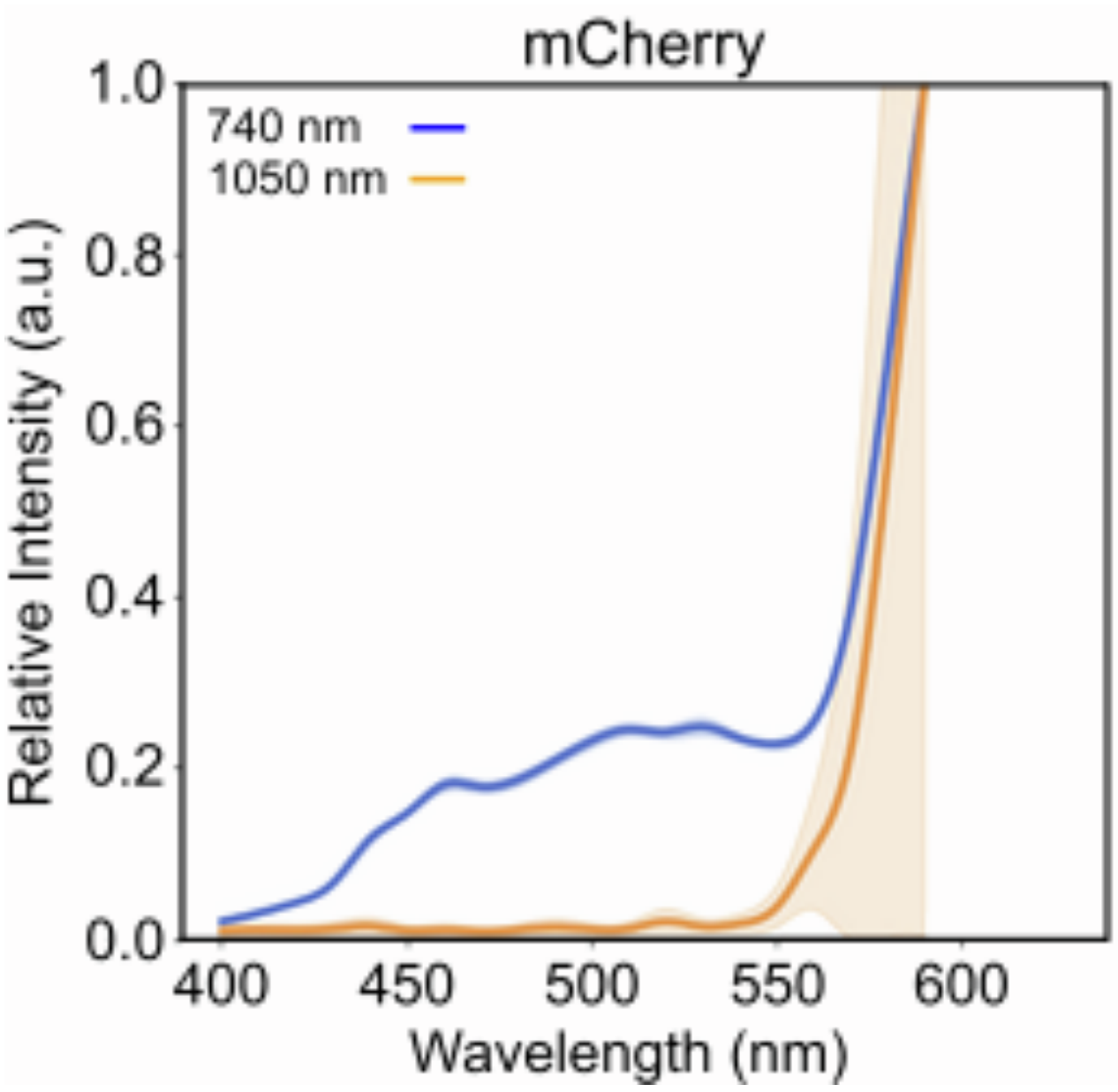

Figure S7: Two-photon emission spectra of mCherry at 740 nm and 1050 nm.
250 nM solution of mCherry in L-15 was excited using 740 nm (blue) and 1050 nm (orange). Values are averaged across an image and normalized to the maximum value. The shaded area represents standard deviation.

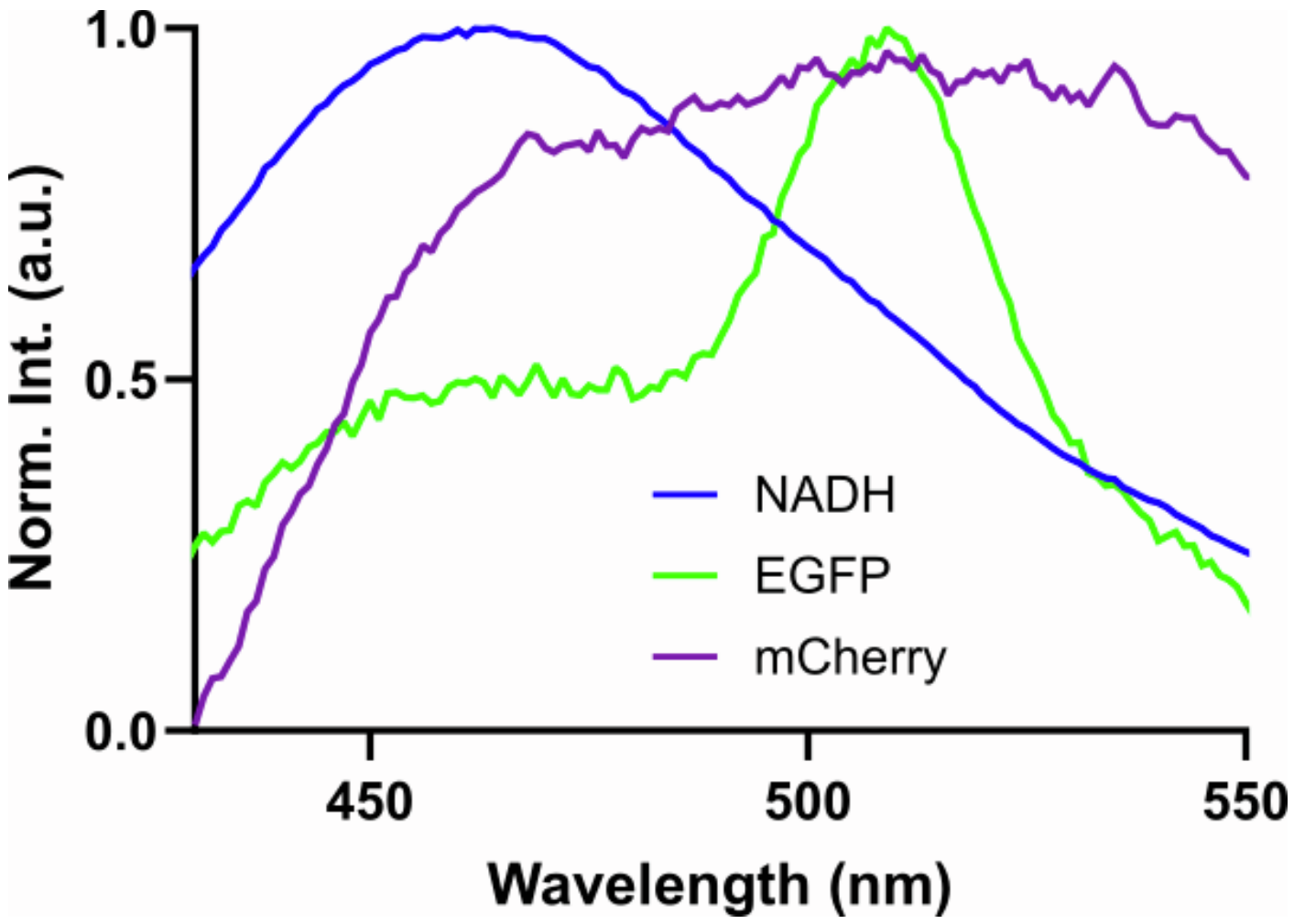

Figure S8: Emission spectra of fluorescent proteins under OPE/UV 370 nm excitation acquired on fluorescence plate reader.

Recombinant proteins, EGFP at 500 nM (green) and mCherry at 1 μM (magenta), and for comparison NADH at 10 mM (blue) were measured in L-15.

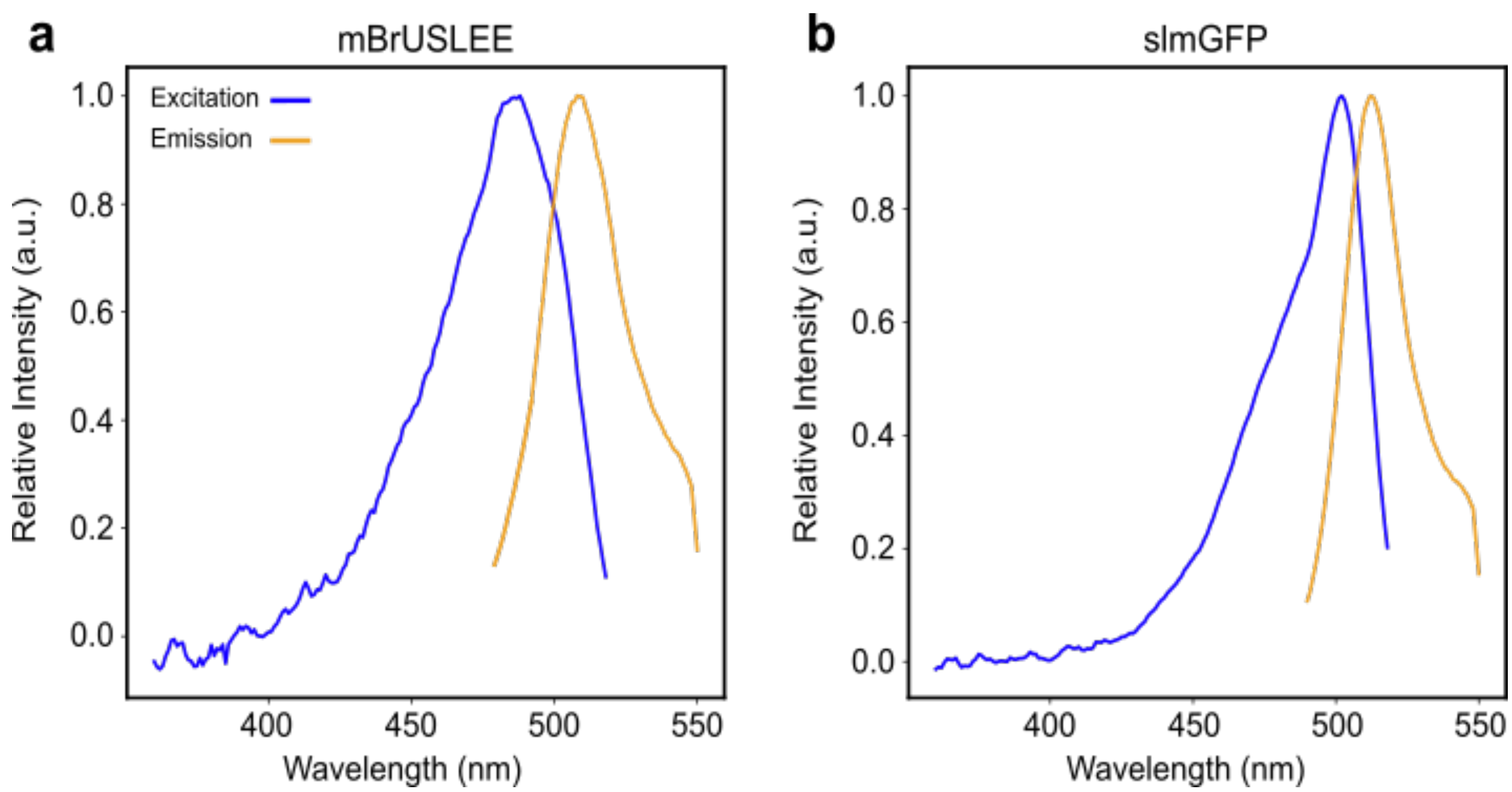

Figure S9: One-photon excitation spectra of short fluorescence lifetime FPs
Fluorescent proteins expressed in U2OS cells as H2B fusions. Blue line is the excitation spectrum, and the yellow line is the emission spectrum.
a mBrUSLEE
b slmGFP
Spectra were acquired in glass bottom 96 well plates on a fluorescence plate reader. Lines represent averages of multiple repeats.

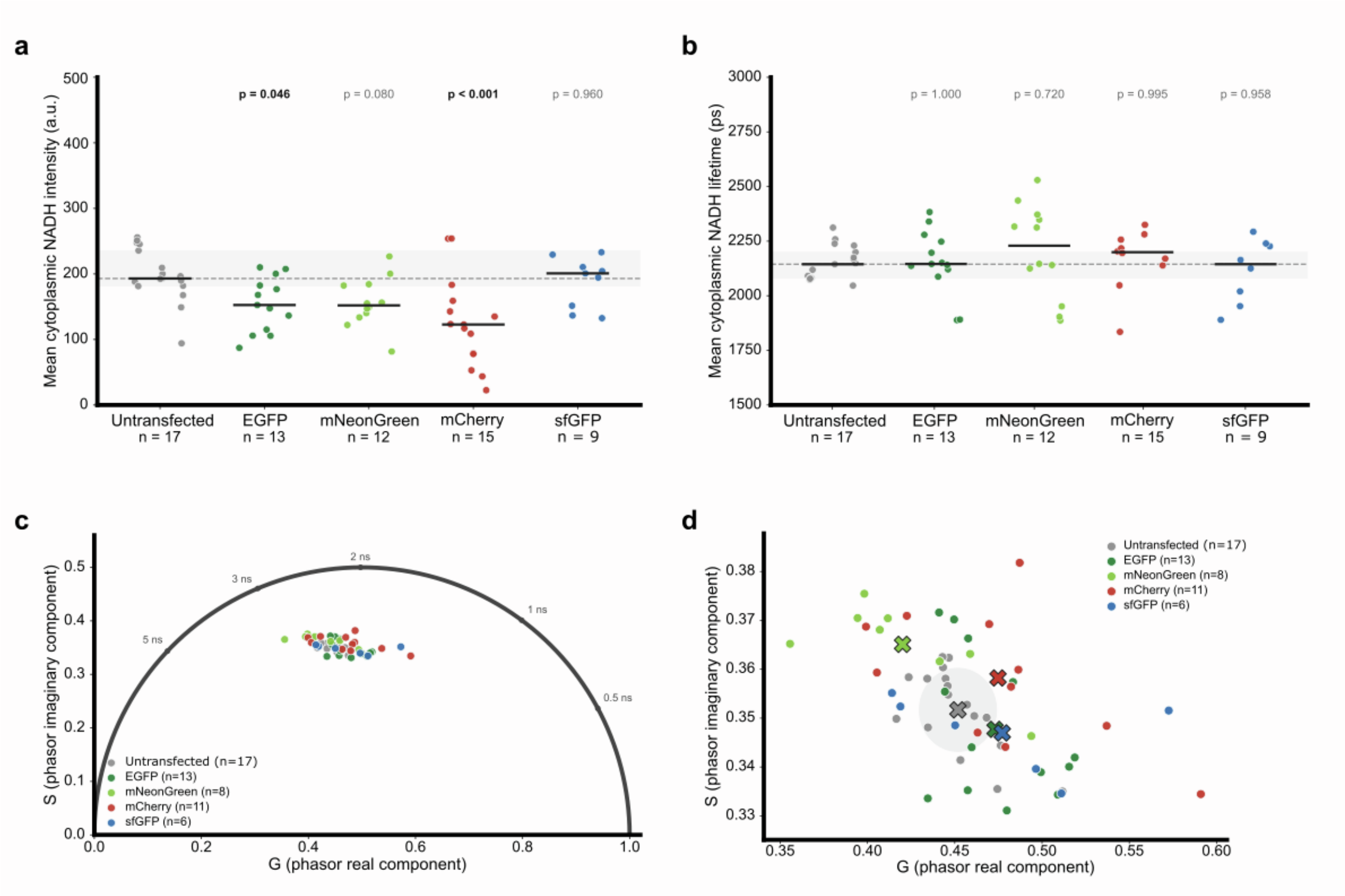

Figure S10: Cytoplasmic NADH measurements in cells expressing nuclear-localized fluorescent proteins.

a Mean cytoplasmic NADH intensity in untransfected cells (n = 17) and cells expressing H2B-tagged EGFP (n = 13), mNeonGreen (n = 12), mCherry (n = 15), or sfGFP (n = 9). Intensity differed among groups (one-way ANOVA, $F(4, 61) = 5.48$, $P < 0.001$).

b Mean cytoplasmic NADH fluorescence lifetime in untransfected cells (n = 17) and cells expressing H2B-tagged EGFP (n = 13), mNeonGreen (n = 12), mCherry (n = 15), or sfGFP (n = 9). No overall difference in lifetime was detected (one-way ANOVA, $F(4, 55) = 0.53$, $P = 0.72$).

In a and b, each circle represents one cell, and short horizontal bars indicate group medians. The dashed line and grey band indicate the median and interquartile range, respectively, of the untransfected control. Dunnett's multiple-comparisons test was used for comparisons with untransfected cells; adjusted p-values are shown above the groups.

c Full phasor distribution for untransfected cells (n = 17) and cells expressing H2B-tagged EGFP (n = 13), mNeonGreen (n = 8), mCherry (n = 11), or sfGFP (n = 6). The solid semicircle denotes the universal semicircle, with the positions of selected single-exponential lifetimes indicated.

d Magnified view of the region occupied by the phasor measurements. Small circles represent individual cells, large X symbols indicate group centroids, and the shaded grey ellipse denotes the +/-1 SD region of the untransfected group. G and S denote the real and imaginary phasor

components, respectively. Intensity is reported in arbitrary units (a.u.) and lifetime in picoseconds (ps).

Table S1: Representative two-photon excitation wavelengths and fluorescence detection windows used for NADH-based metabolic imaging in published studies.

Reference ID	Reference (year)	Sample context	Excitation (nm)	Detection window (nm)	Link
**1**	York 2019	GFP interference	750	460/50	pmc.ncbi.nlm.nih.gov
**2**	Schroeder 2020	Glioblastoma stem cells	740	450/70	pmc.ncbi.nlm.nih.gov
**3**	Baraghis 2011	Cortical tissue	740	460±25	pmc.ncbi.nlm.nih.gov
**4**	Lam 2016	Valve interstitial cells	755	460±20	pmc.ncbi.nlm.nih.gov
**5**	Chen 2021	Brain tissue	780	447/60	thno.org
**6**	Azzarello 2024	Islet α/β cells	740	420–460	pmc.ncbi.nlm.nih.gov
**7**	Sameni 2016	Huntington's disease cells	740	442/46	pmc.ncbi.nlm.nih.gov
**8**	Bragin 2015	Brain microvasculature	740	425–475	pmc.ncbi.nlm.nih.gov
**9**	Ranjit 2019	MEF cells	740	460/40	pmc.ncbi.nlm.nih.gov
**10**	Niederschweiberer 2021	Primary Hippocampal Neurons	730	460/60	frontiersin.org
**11**	Paillon 2024	T lymphocytes	740	460/80	pmc.ncbi.nlm.nih.gov
**12**	Huang 2002	Redox ratio imaging	800/750	410–490	pmc.ncbi.nlm.nih.gov
**13**	Snyder 2023	HIV-infected cells	780	460/55	pmc.ncbi.nlm.nih.gov

Supporting References

1. York EM, Weilinger NL, LeDue JM, MacVicar BA. Green fluorescent protein emission obscures metabolic fluorescent lifetime imaging of NAD(P)H. Biomed Opt Express. 2019;10(9):4381–4394. https://doi.org/10.1364/BOE.10.004381

2. Schroeder AB, Pointer KB, Clark PA, Datta R, Kuo JS, Eliceiri KW. Metabolic mapping of glioblastoma stem cells reveals NADH fluxes associated with glioblastoma phenotype and survival. J Biomed Opt. 2020;25(3):036502. https://doi.org/10.1117/1.JBO.25.3.036502

3. Baraghis E, Devor A, Fang Q, Srinivasan VJ, Wu W, Lesage F, et al. Two-photon microscopy of cortical NADH fluorescence intensity changes: correcting contamination from the hemodynamic response. J Biomed Opt. 2011;16(10):106003. https://doi.org/10.1117/1.3633339

4. Lam NT, Muldoon TJ, Quinn KP, Rajaram N, Balachandran K. Valve interstitial cell contractile strength and metabolic state are dependent on its shape. Integr Biol (Camb). 2016;8(10):1079–1089. https://doi.org/10.1039/c6ib00120c

5. Chen D, Nauen DW, Park HC, Li D, Yuan W, Li A, et al. Label-free imaging of human brain tissue at subcellular resolution for potential rapid intra-operative assessment of glioma surgery. Theranostics. 2021;11(15):7222–7234. https://doi.org/10.7150/thno.59244

6. Azzarello F, Carli F, De Lorenzi V, Tesi M, Marchetti P, Beltram F, et al. Machine-learning-guided recognition of α and β cells from label-free infrared micrographs of living human islets of Langerhans. Sci Rep. 2024;14:14235. https://doi.org/10.1038/s41598-024-65161-7

7. Sameni S, Syed A, Marsh JL, Digman MA. The phasor-FLIM fingerprints reveal shifts from OXPHOS to enhanced glycolysis in Huntington Disease. Sci Rep. 2016;6:34755. https://doi.org/10.1038/srep34755

8. Bragin DE, Statom GL, Hagberg S, Nemoto EM. Increases in microvascular perfusion and tissue oxygenation via pulsed electromagnetic fields in the healthy rat brain. J Neurosurg. 2015;122(5):1239–1247. https://doi.org/10.3171/2014.8.JNS132083

9. Ranjit S, Malacrida L, Stakic M, Gratton E. Determination of the metabolic index using the fluorescence lifetime of free and bound nicotinamide adenine dinucleotide using the phasor approach. J Biophotonics. 2019;12(11):e201900156. https://doi.org/10.1002/jbio.201900156

10. Niederschweiberer MA, Schaefer PM, Singh LN, Lausser L, Bhosale D, Hesse R, et al. NADH fluorescence lifetime imaging microscopy reveals selective mitochondrial dysfunction in neurons overexpressing Alzheimer's disease-related proteins. Front Mol Biosci. 2021;8:671274. https://doi.org/10.3389/fmolb.2021.671274

11. Paillon N, Ung TPL, Dogniaux S, Stringari C, Hivroz C. Label-free single-cell live imaging reveals fast metabolic switch in T lymphocytes. Mol Biol Cell. 2024;35(1):ar11. https://doi.org/10.1091/mbc.E23-01-0009

12. Huang S, Heikal AA, Webb WW. Two-photon fluorescence spectroscopy and microscopy of NAD(P)H and flavoprotein. Biophys J. 2002;82(5):2811–2825. https://doi.org/10.1016/S0006-3495(02)75621-X

13. Snyder GA, Kumar S, Lewis GK, Ray K. Two-photon fluorescence lifetime imaging microscopy of NADH metabolism in HIV-1 infected cells and tissues. Front Immunol. 2023;14:1213180. https://doi.org/10.3389/fimmu.2023.1213180
