# Supplementary material for: Bright probes, blurred metabolism: Navigating fluorescent protein cross-excitation in NADH FLIM
Source: Biophys Rep (N Y). 2026 Oct 20;6(4):100294. doi: 10.1016/j.bpr.2026.100294 (PMC13769086; doi:10.1016/j.bpr.2026.100294)
Supplement: Supplementary file 2 [file mmc2.pdf]

Biophysical Reports

ARTICLE

Bright probes, blurred metabolism: Navigating fluorescent protein cross-excitation in NADH FLIM

Janielle Cuala,[1,2,10] Oscar Alberto de la Fuente,[3,4,10] Lynne Cherchia,[3,5] Yuanzhong Pan,[5] Tvisha Singh,[6] Chris Deng,[6,7] Andrea Velazquez,[6] Kate White,[6] Senta K. Georgia,[2] Steve A. Kay,[5] Scott E. Fraser,[3,8] and Falk Schneider[3,6,9,*]

[1]Department of Medical Biophysics, Keck School of Medicine, University of Southern California, Los Angeles, CA, USA; [2]Center for Endocrinology, Diabetes, and Metabolism, The Saban Research Institute, Children's Hospital Los Angeles, Los Angeles, CA, USA; [3]Translational Imaging Center, Michelson Center for Convergent Bioscience, University of Southern California, Los Angeles, CA, USA; [4]Department of Development, Stem Cells and Regeneration, Keck School of Medicine, University of Southern California, Los Angeles, CA, USA; [5]Alfred E. Mann Department of Biomedical Engineering, Viterbi School of Engineering, University of Southern California, Los Angeles, CA, USA; [6]Dana and David Dornsife College of Letters, Arts and Sciences, University of Southern California, Los Angeles, CA, USA; [7]Shu Chien-Gene Lay Department of Bioengineering, Jacobs School of Engineering, University of California, San Diego, San Diego, CA, USA; [8]Biohub, Redwood City, CA, USA; and [9]Directorate of Biomedical Sciences, Warwick Medical School, University of Warwick, Coventry, UK
[10]These authors contributed equally

ABSTRACT Fluorescence lifetime imaging microscopy (FLIM) of endogenous NAD(P)H enables the label-free assessment of cellular metabolic state. Although metabolic imaging is increasingly combined with fluorescent protein (FP) reporters to enhance biological specificity, the potential crosstalk between the intrinsic and extrinsic labels remains ill-defined. Here, we systematically evaluate crosstalk from FPs in metabolic FLIM using phasor analysis of two-photon fluorescence microscopy. The results clearly show that many widely used FPs are excited under the conditions used for NAD(P)H imaging; they emit blue-shifted, short-lifetime fluorescence that can interfere with imaging NAD(P)H metabolic signatures. This overlap persists across excitation wavelengths and FP classes, posing a significant challenge for multiplexed metabolic imaging. This cautionary tale argues against unvalidated multiplexing strategies in metabolic FLIM studies. Our study aims to identify acceptable imaging partners, offer a pipeline for assaying potential crosstalk, and provide practical guidance for experimental design.

WHY IT MATTERS Fluorescence lifetime imaging of NAD(P)H autofluorescence is a powerful, label-free approach to map cellular metabolism in living tissues. A growing number of studies combine it with fluorescent protein (FP) reporters to simultaneously identify specific cell types or subcellular compartments. This study reveals that many FPs, spanning the visible spectrum, are unexpectedly excited under NAD(P)H imaging conditions. Commonly used green, yellow, and red variants produce short-lifetime, blue-shifted fluorescence that directly overlaps with metabolic NAD(P)H signals. This cross-excitation can be falsely interpreted as a shift in cellular metabolic state, posing a significant risk to multiplexed metabolic imaging studies. Our work establishes a pipeline to assess this risk and provides a practical framework to guide experimental design for multiplexed metabolic FLIM.

INTRODUCTION

Cellular metabolism rapidly adapts to changes in physiology, stress, and disease and is an indicator of cellular state. The ability to monitor metabolic states and their transitions therefore represents a powerful approach to study cellular function and to diagnose disease.[1,2] Cancer cells, for instance, shift their metabolism toward glycolysis to sustain high ATP demand, known as the Warburg effect.[3,4] In the context of COVID-19 infection, cells such as monocytes and macrophages undergo metabolic changes during infection from the energy-efficient process of oxidative phosphorylation (OXPHOS) to glycolysis, which has been implicated in enhanced SARS-CoV-2 replication.[5] In diabetes, hyperglycemia reduces pancreatic β-cell mitochondrial metabolism, observed as an upregulation of glycolysis-related genes and a reduction of OXPHOS.[6] Together, these examples

Submitted June 15, 2026, and accepted for publication October 16, 2026.

*Correspondence: falk.schneider@warwick.ac.uk

Editor: Erdinc Sezgin.

https://doi.org/10.1016/j.bpr.2026.100294

© 2026 The Author(s). Published by Elsevier Inc. on behalf of Biophysical Society.
This is an open access article under the CC BY license (http://creativecommons.org/licenses/by/4.0/).

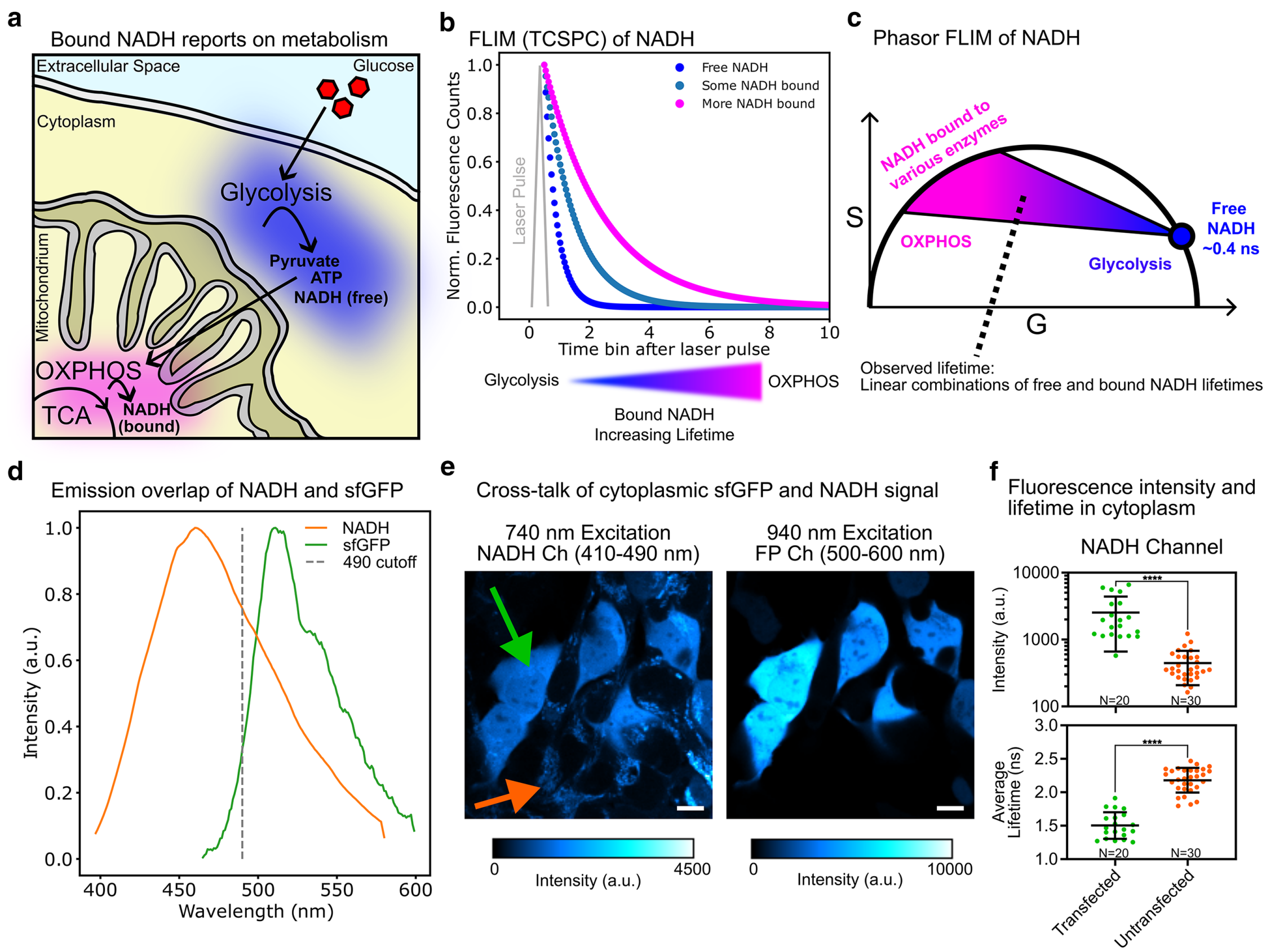

Figure 1. Metabolic imaging with FLIM. (a) Cartoon illustrating subcellular localization of glycolysis and oxidative phosphorylation (OXPHOS), as well as resulting bound or free NADH. (b) Exponential decays illustrating time-correlated single-photon counting decays of free NADH (assuming a lifetime of 0.4 ns) and bound NADH (longer lifetimes). (c) Cartoon of a phasor plot for metabolic imaging. Monoexponential lifetimes fall on the unity circle. Free NADH has a monoexponential lifetime of 0.4 ns. NADH bound to various enzymes (mixed with free NADH) can be found on the metabolic trajectory indicated by blue to magenta shading. Shorter lifetimes toward free NADH (blue) indicate glycolysis, whereas longer lifetimes (magenta) indicate OXPHOS as the prevalent metabolic state in a measurement. (d) Normalized fluorescence emission spectra of NADH and sfGFP-H2B expressed in HEK cells. Dashed gray line indicates a commonly used cutoff filter to avoid recording FAD fluorescence signal and/or other fluorescent species. (e) Laser scanning two-photon excitation microscopy images of HEK cells expressing cytoplasmic sfGFP. With 740 nm excitation, NADH detection at 410–490 nm (left), and FP detection at 500–600 nm (right), mitochondria and cytoplasmic sfGFP can be observed. Arrows indicate transfected and untransfected cells (green and orange, respectively). With 940 nm excitation and FP detection, sfGFP can be observed. Scale bars: 10 μm. (f) Fluorescence intensity and average fluorescence lifetime of the cytoplasm area segmented from the transfected and untransfected cells. Every dot represents an area segmented from a single cell. Horizontal bars indicate mean values and vertical lines indicate standard deviations (****$p \leq 0.0001$, from two-tailed t test).

highlight metabolism as both a readout and a driver of disease progression.

Robustly measuring changes in cellular metabolism is of critical importance to understanding cellular physiology. Biochemical approaches can be used to monitor biomarkers and extracellular acidification[7]; however, such methods rely on homogeneous samples or dissociated tissues. Microscopy methods, instead, enable noninvasive observation of changes to metabolism in intact and living samples. To this end, a variety of biosensors for visualizing specific metabolites have been developed.[8,9] For instance, PercevalHR can be used to look at changes in ATP/ADP ratio,[10] FapR-NLuc to observe malonyl-CoA in liver fatty acid metabolism,[11,12] TINGL to monitor glucose levels,[13] and Peredox to report on NADH/NAD+ ratio.[14] This nonexhaustive list shows that these sensors become increasingly powerful, yet a biosensor's potential can be limited by experimental variables such as expression level, detection efficiencies, varying dissociation constants, or changes within the local physicochemical environment.[15,16]

Metabolic imaging exploits the autofluorescence of endogenous metabolic compounds and thus presents a label-free remedy. Specifically, NAD(P)H and FAD are intermediate substrates in metabolism that can be excited using ultraviolet (UV) light or two-photon excitation (TPE).[17,18] Their concentration and interaction with enzymes of metabolic pathways (Figure 1a) are important factors in assessing the metabolic state of cells and tissues.[19] In the following, NADH will be used to collectively refer to both NADH and NADPH. Importantly, the interaction of NADH with enzymes

causes only minor changes to its emission spectrum but results in measurable changes to its fluorescence lifetime, which is the average time a fluorophore spends in the excited state. This is used in fluorescence lifetime imaging microscopy (FLIM) as an additional means to generate contrast in the image. The fluorescence lifetime of free NADH is $\sim$0.4 ns and increases when bound to different enzymes to 1–5 ns (see illustration in Figure 1b). The longer the lifetime, the more NADH is bound to proteins, specifically to enzymes of the OXPHOS pathway.[18,20] In this way, changes to NADH lifetime can be used as a biophysical readout to report on shifts in cellular metabolism from glycolysis to OXPHOS, or vice versa.[21]

Fluorescence lifetime data can be acquired using one-photon excitation (OPE) or TPE. Due to its superior signal/noise ratio (SNR), higher penetration depth, and reduced phototoxicity compared with UV exposure, TPE is commonly used to study free and bound NADH levels.[17,21] It can be combined with FLIM acquisitions in the frequency domain using excitation phase modulation or using pulsed excitation with time-correlated single photon counting (TCSPC), leading to fluorescence decay curves per pixel (Figure 1b). For the latter, decay data are typically fitted by exponential models wherein the decay time represents the lifetime. In the case of metabolic imaging data, fitting can be a difficult task because mixtures of species (free NADH and NADH bound to various enzymes) are present and vary spatially. The phasor approach to fluorescence lifetime presents a remedy, as it converts the complex modulation or decay data to a projection on a 2D plane (Figure 1c).[22] Every pixel in the FLIM dataset has an intensity value, an average lifetime value, and two phasor coefficients, usually labeled G and S. The phasor approach does not require fitting and thus does not make assumptions about the number of components present. It does indicate if the decay is mono- or multi-exponential, as single-component data map onto the phasor circumference, whereas multicomponent data fall within the circle. Furthermore, it helps deal with low-SNR data and explore the number of different lifetime components in the sample.[23–25] Phasor analysis is becoming the go-to tool for metabolic imaging.

Label-free FLIM can be combined with fluorescent labels to increase the information content and provide cellular context. For example, labeling a certain population of cells (e.g., cancerous, infected, or diabetic) with an additional fluorophore can provide mechanistic insights. The fluorophores of choice are typically fluorescent proteins (FPs), which can be conveniently expressed in the cells of interest. Metabolic imaging of NADH has been reported in combination with imaging of, for example, GFP,[21] EGFP,[26,27] or mCherry.[28] Fluorescence emission from the FPs and NADH was reported to be separated using stringent emission filters. However, concerns regarding cross-excitation of NADH and FPs, signal spillovers, and blue-shifted FP emission have been raised.[29] The photophysics of FPs are complicated, depending on the exact sequence of the FP and the cellular environment, and might vary between OPE and TPE.[30–33] Thus, different versions of EGFP could have completely different fluorescence properties. For instance, pioneering work on wild-type GFP excited-state dynamics has shown that the protonated chromophore (often referred to as the A-state) can be excited around 400 nm in OPE and 800 nm in TPE.[34,35] This can result in rapid internal proton transfer, causing fluorescence around 510 nm, or, more rarely, in direct A-state emission around 460 nm with lifetimes shorter than 1 ns (which would overlap with NADH fluorescence). The anionic form (B-state) can be excited around 480 nm (OPE) or around 930–950 nm (TPE), resulting in fluorescence around 510 nm. The S65T mutation, among others in EGFP, has been shown to alter the internal hydrogen-bonding network. That suppresses internal proton transfer and shifts the chromophores' equilibrium toward the anionic form, reducing excitation of the A-state.[36,37] The excitation/emission properties of different FPs at 740 nm (TPE) and in the physicochemical environment of the cell can be complex and difficult to predict.[38] Importantly, due to the dim nature of NADH fluorescence (low quantum yield) as compared to FPs, any crosstalk needs to be ruled out and can be particularly difficult to account for.

In this work, we systematically address the issue of metabolic imaging of NADH in the presence of another fluorescently labeled species. We employ the phasor approach to separate NADH from FP signals and reveal blue-shifted fluorescence with short lifetimes for multiple FPs, which can potentially pollute the metabolic imaging of NADH. We demonstrate that all tested FPs show overlap with the metabolic imaging space mapped out by drug and glucose treatments. Overall, we aim to provide comprehensive guidelines for performing metabolic imaging in context.

MATERIALS AND METHODS

Reagents

Common reagents used in this study are summarized in Table 1.

Plasmid design

All plasmids used in this study are summarized in Table 2. For the TPE crosstalk assay, all FPs have

Table 1. Reagents used in this study

Reagent	Source and cat. no.
μ-Slide 8-well slide	ibidi, #80807
96-well TC-treated imaging microplate	Corning, #353219
24-well glass-bottom plate	Cellvis, #NC0397150
In-Fusion Snap Assembly	Takara, #638947
DMEM	Corning, #10-013-CV
FBS	Thermo Fisher Scientific, #A5256701
Penicillin/streptomycin	Thermo Fisher Scientific, #15140122
L-glutamine	Sigma-Aldrich, #G7513
Phenol red-free Leibovitz's L-15	Thermo Fisher Scientific, #21083027
Lipofectamine	Thermo Fisher Scientific, #L30008
Recombinant EGFP	OriGene, #TP790050
Recombinant mCherry	Abcam, #ab199750
NADH	Sigma-Aldrich, #481913-1GM
D-glucose	Gibco, #A24940-01
Antimycin A	Thermo Fisher Scientific, #J63522.MA, Lot# T21J051
Rotenone	Sigma-Aldrich, #557368

been fused to human H2B, creating a library of constructs to test. The FP amino acid sequences have been checked against the respective entry on FPbase.org.[40] The H2B sequence has been PCR amplified from a pCS2+-mCherry-H2B plasmid (kind gift from Le Trinh, USC). PCRs were performed using LA Taq (#RR002A) according to the manufacturer's protocol. A short linker sequence has been introduced to separate FP and H2B: GSGPVATGSG. Cloning has been performed using Takara In-Fusion Snap Assembly (#638947) according to the manufacturer's protocol. We used a pCS2+ plasmid (kind gift from Le Trinh, USC) containing a CMV promoter for expression in mammalian cells as a backbone. sfGFP-N1 was a gift from Michael Davidson and Geoffrey Waldo (Addgene plasmid #54737; http://n2t.net/addgene:54737; RRID:Addgene_54737). FPs were obtained as gene blocks with flanking regions for In-Fusion (synthesized by IDT) or amplified by PCR. The enhancer vhh sequence was taken from prior studies.[41,42] A T2A sequence (EGRGSLLTCGDVEENPGP) has been used for equimolar expression of vhh and FP-H2B.[43]

Cell culture and transfection

U2OS cells were obtained from the American Type Culture Collection and cultured in DMEM (Corning, #10-013-CV) supplemented with 10% fetal bovine serum (Thermo Fisher Scientific, #A5256701), 1% penicillin/streptomycin (Thermo Fisher Scientific, #15140122), and 1% L-glutamine (Sigma-Aldrich, #G7513). Cells were grown to approximately 80% confluency before being seeded onto an 8-well μ-Slide (ibidi, #80807). 24 h after seeding, cells were transfected using Lipofectamine 3000 and P3000 reagents (Thermo Fisher Scientific, #L30008) according to the manufacturer's protocol. 24–48 h after transfection, cell culture medium was replaced with prewarmed, phenol red-free Leibovitz's L-15 medium (Thermo Fisher Scientific, #21083027) prior to image acquisition.

Plate reader: Spectra

U2OS cells were seeded onto a black 96-well plate (Corning, #353219) or a black glass-bottom 24-well plate (Cellvis, #NC0397150) and transfected as described above. 24 h after transfection, cell culture medium was replaced with prewarmed L-15, followed by a spectra scan on a CLARIOstar Plus microplate reader using CLARIOstar MARS software. Mean intensity values from the exported plate reader CSV files were corrected per condition to the background fluorescence emitted by either the medium-only blank for protein solution measurements or untransfected samples for cell culture measurements. For plotting, a per-FP average over 3–6 technical replicates of the corrected data was obtained, and the resulting values were normalized to FP's maximum values across the emission scan. Smoothing was performed using custom Python code: https://github.com/janiellecuala/Multiplexing-NADH-and-Fluorescent-Proteins-in-Metabolic-Imaging.

TPE microscopy

FLIM was performed on an SP8 DIVE FALCON spectral multi-photon FLIM microscope (Leica Microsystems, Germany) using an HC PL AP 63×/1.2 NA motCorr water-immersion objective (Leica, #11506361). NAD(P)H was excited with a Spectra-Physics Insight 3X ultrafast infrared (IR) laser at 0.8 mW average power, with 20–40 frame accumulations per optical section. Excitation was performed as indicated: 740 nm was used for NADH excitation, and 940 nm was used for fluorophore excitation, except for stagRFP and recombinant mCherry, which were excited at 1,050 nm. Images at different excitation wavelengths were acquired sequentially (identification of transfected cells and crosstalk assessment). Most images were collected at 512 × 512 px and 2.0–2.5 zoom, resulting in a final pixel size of 144 nm/px. Spectral detection was performed using SMD-HyD detectors in nondescanned (NDD) configuration, with detection windows as indicated: 410–490 nm for NADH, 500–600 nm for green/yellow FP detection, and 600–700 nm for red FP detection. Where indicated, lambda scans were performed by varying the excitation or emission

Table 2. Plasmids used in this study

Plasmid	FP (FPbase identifier)	More information
pCS2+-mNeonGreen	mNeonGreen, https://www.fpbase.org/protein/mneongreen/	Cytoplasmic FP
sfGFP-N1	superfolder GFP, sfGFP, https://www.fpbase.org/protein/superfolder-gfp/	Addgene plasmid 54737, cytoplasmic FP
pCS2+-mNG-H2B	mNeonGreen, https://www.fpbase.org/protein/mneongreen/	N/A
pCS2+-sfGFP-H2B	sfGFP, https://www.fpbase.org/protein/superfolder-gfp/	N/A
pCS2+-mEGFP-H2B	mEGFP, https://www.fpbase.org/protein/megfp/	N/A
pCS2+-avGFP-H2B	wild-type GFP, avGFP, https://www.fpbase.org/protein/avgfp/	N/A
pCS+-mCitrine-H2B	mCitrine, https://www.fpbase.org/protein/mcitrine/	N/A
pCS2+-mCherry-H2B	mCherry, https://www.fpbase.org/protein/mcherry/	N/A
pCS2+-stagRFP-H2B	super-TagRFP, stagRFP, https://www.fpbase.org/protein/super-tagrfp/	N/A
pCS2+-mBrUSLEE-H2B	bright ultimately short lifetime enhanced emitter, mBrUSLEE, https://www.fpbase.org/protein/bruslee/	Identical amino acid sequence to FP base entry but added A206K mutation
pCS2+-slmGFP-H2B	short-lifetime monomeric GFP, slmGFP	slmGFP sequence[39] was a kind gift from Michael Knop, ZMBH
pCS2+-StayGoldE138D-H2B	StayGoldE138D, https://www.fpbase.org/protein/staygold-e138d/	N/A
pCS2+-mStayGold2-H2B	mStayGold2, https://www.fpbase.org/protein/mstaygold2/	N/A
pCS2+-enhancerVhh-T2A-mEGFP-H2B	mEGFP, https://www.fpbase.org/protein/megfp/	N/A
pCS2+-enhancerVhh-T2A-avGFP-H2B	avGFP, https://www.fpbase.org/protein/avgfp/	N/A

wavelength by 10 nm increments. *In vitro* images were collected with 20 frame repetitions at 128 × 128 px, using a zoom factor of 5, which resulted in a final pixel size of 290 nm/px.

Metabolic space and *in vitro* measurements

Glucose-stimulated experiments were performed with solutions made from Krebs-Ringer Buffer solution to make 2.8 mM low glucose (LG), 16.7 mM high glucose (HG) (Gibco, #A24940-01), and HG with 1 μM rotenone (Sigma-Aldrich, #557368) and with 1 μg/mL antimycin-A (HG+drug) (Thermo Fisher Scientific, #J63522.MA). All samples were starved in LG solution for 1 h, then the LG condition was imaged. For the HG condition, the LG solution was replaced with HG solution for 30 min, then imaged. Finally, for the HG+drug condition, the HG solution was then replaced with the HG+drug solution for 30 min, then imaged.

For *in vitro* measurements, recombinant proteins and NADH were imaged in L-15 on μ-Slide 8-well coverslips. EGFP (OriGene, #TP790050) was imaged at a concentration of 150 nM, mCherry (Abcam, #ab199750) was imaged at 250 nM, and NADH (Sigma-Aldrich, #481913-1GM) was imaged at 200 μM.

Data processing and analysis

FLIM images were preprocessed in the Leica LAS X software using the phasor FLIM module. Pixels were binned 3 × 3, and a threshold of 10 photons was applied (i.e., all pixels with less than 10 photons are disregarded). Phasor coordinates were filtered using the built-in wavelet filter. Fast FLIM images were exported along with intensity and G/S images. Thus, for each detector, a total of 4 images were exported: from channels 0–3, representing intensity, lifetime, and G and S coordinates, respectively. Any pseudocolored lifetime images were directly exported from LAS X. For pseudocolored metabolic images, the LAS X Metabolic Arc module was applied to the phasors, generating isolines representing lifetimes in phasor space that can be generated by different combinations of long and short lifetimes. Free NADH lifetime was set to 0.4 ns. Thus, the isolines/arcs can be thought of as visualizing all locations on the phasor plot with the same ratio of bound to free NADH.

TIFF files were processed using Fiji/Image J.[44] Regions of interest (ROIs) for transfected nuclei were drawn based on the intensity image of the

fluorophore; untransfected nuclei and cytoplasm ROIs were selected based on the NADH intensity image. Separate CSV files were exported with the mean values for intensity, lifetimes, and G and S coordinates. A macro for automated processing in Fiji can be found on our GitHub.

For TPE and emission scan plots, mean intensity values from the exported CSV file for the ROIs from 680 to 780 nm were plotted with the Python code found on our GitHub. Values were normalized first to excitation laser power, then each individual nucleus and cytoplasm to the highest intensity value detected in the same ROI across the excitation scan, effectively setting the emission peak to 1 (per cell). Finally, the individual max normalized cell traces were averaged across cells/repeats, generating one trace per FP. For metabolic trajectory boundary analysis, mean G and S coordinates were plotted with GraphPad Prism 10. For each experiment, at least 3 biological replicates (i.e., different imaging sessions with various sets of cells) were performed, and at least 5 technical replicates (i.e., number of transfected, untransfected, and cytoplasmic ROIs) were performed for each FP or condition.

RESULTS

We set out to multiplex NADH and FPs on a TPE setup. We began by assessing GFPs, as the OPE emission spectra of sfGFP, wild-type GFP from *Aequoria victoria* (avGFP), mEGFP, and mNeonGreen (mNG) based on our own (Figure S1) and published data[40,45] could be reasonably separated from NADH, allowing for the use of further red-shifted probes to label additional biological structures. The combination of sfGFP and NADH, in particular, would represent a powerful pair for metabolic imaging in the context of diabetes research due to the existence of the c-peptide-sfGFP (CpepSfGFP) mouse line.[46] The OPE emission spectra of NADH and sfGFP show only minor overlap (Figure 1d). Crosstalk from NADH to the FP detection channel is less of a concern due to the difference in brightness of the fluorophores, but crosstalk from sfGFP to the NADH channel would pose an issue. However, stringent filters, different excitation wavelengths, and dedicated tracks may allow simultaneous imaging. When employing TPE at 940 nm on cells transfected with sfGFP, we can clearly identify the FP fluorescence (in the FP emission channel, 500–600 nm). When using 740 nm for excitation, we do not expect excitation of sfGFP. However, crosstalk becomes apparent in fluorescence images as cytoplasmic and nucleoplasmic haze, whereas untransfected cells show only mitochondrial signal from NADH autofluorescence (Figure 1e). At 740 nm excitation, the raw intensity is about five times higher in transfected versus untransfected cells, and a decrease in average fluorescence lifetime from 2.2 to 1.5 ns is observed (Figure 1f), indicative of a shift in metabolic state toward glycolysis. As sfGFP shows fluorescence emission overlap with NADH emission, we assessed cytoplasmic mNG in the same way and observed distinct cytoplasmic haze in transfected cells, albeit less pronounced than for sfGFP (Figure S2). To assess green/yellow FP emission in the absence of the added complexity of cells, we recorded emission spectra of recombinant EGFP excited at 740 and 940 nm (TPE) and observed extension of the emission peak to shorter wavelengths under 740 nm TPE (Figures S3 and S4). Taken together, these experiments lead us to believe that some GFPs can be excited using 740 nm and show undesired fluorescence properties such as blue-shifted emission and altered lifetime. This may cause crosstalk with the NADH signal, potentially biasing metabolic imaging.

These issues are challenging to assess in live cells expressing cytoplasmic FPs due to spatial overlap with NADH fluorescence (cytoplasmic and mitochondrial). To investigate crosstalk under cellular/physiological conditions, we designed an assay with nuclear-localized FP (conjugated to H2B) to better separate NADH and FP fluorescence, as only minimal signal from free NADH is expected in the nucleus[47–49] (Figures 2a and S5). We recorded fluorescence sequentially at 940 nm TPE in the NADH channel (410–490 nm) and FP channel (500–600 nm) to identify transfected cells and record typical fluorescence behavior, and then acquired at 740 nm TPE in the NADH and FP channels as before. The resulting four-channel images and respective FLIM data are displayed and analyzed using phasor plots and inform fluorescence properties and crosstalk (Figure 2a). Ideally, the FP should show a near-monoexponential lifetime and map to the phasor circle when excited with 940 nm (emission is expected roughly between 500 and 600 nm based on OPE spectra; Figure S1). Excitation with 740 nm should cause either no fluorescence or only weak fluorescence in the FP channel. NADH autofluorescence should not be observed in the FP channel, and FP fluorescence should not show in the NADH channel. Thus, the four phasor plots can be used to quickly assess potential cross-excitation and cross-talk of FP and NADH and should allow for observation of blue-shifted fluorescence, if present.

Performing the cross-excitation assay on sfGFP-H2B-transfected cells reveals clear nuclear fluorescence from 740 and 940 nm TPE in all four channels (Figure 2b). A low level of background fluorescence from the nuclei in the NADH channel is expected

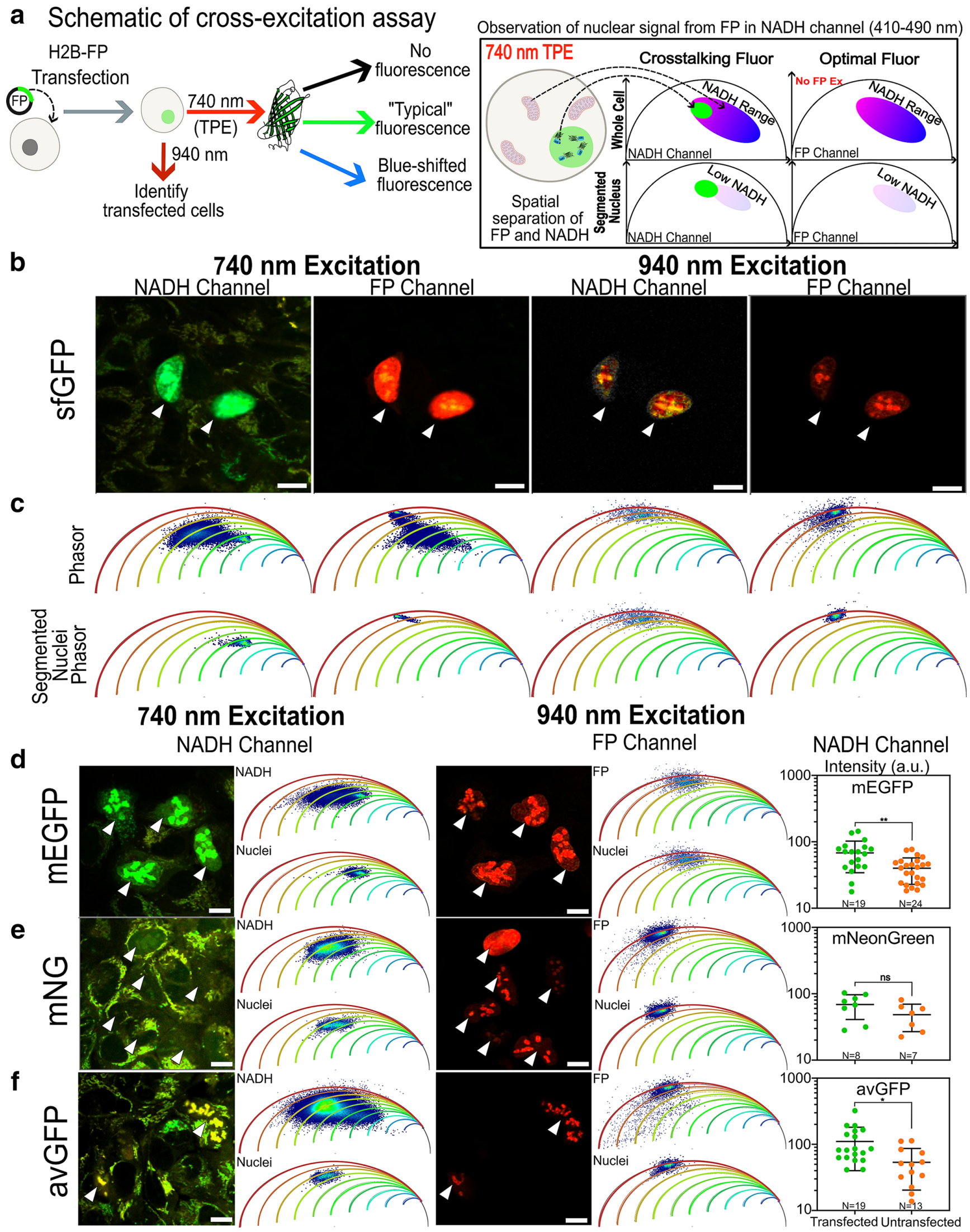

Figure 2. TPE cross-excitation assay using nuclear-localized FPs. (a) Cartoon illustrating experimental setup of TPE cross-excitation assay. Briefly, U2OS cells are transfected with the respective fluorescent protein (FP). Using TPE excitation at 740 nm, the FP can be determined to be crosstalking or optimal based on phasor signal separation of FP and NADH signals. With TPE 940 nm excitation, the identification of transfected nuclei can be confirmed. Ideally, nuclear localization of fluorophore expression minimizes spatial crosstalk between FP and NADH signals. (b and c) TPE cross-excitation assay for sfGFP. (b) Laser scanning TPE microscopy images of U2OS cells expressing nuclear sfGFP with 740 or 940 nm excitation. Detection in the NADH channel range 410–490 nm and FP range 500–600 nm. Arrows indicate the transfected cells with nuclear sfGFP. With 940 nm excitation and FP detection, sfGFP can be observed. Color is based on the phasor plot metabolic arcs below. Scale bars: 10 μm. (c) Phasor plots associated with each image above are shown with metabolic arcs represented with a rainbow LUT. For NADH channel this may represent the percentage of bound NADH, where blue is 0% and red is 100%. The top row represents the NADH channel that includes all pixels from the whole image. The bottom row is the phasor signal from the segmented nuclei of the transfected cells, shown with the white arrows in (b). (d–f) TPE cross-excitation assays. Laser scanning TPE microscopy images of U2OS cells expressing nuclear (d) mEGFP, (e) mNG, and (f) avGFP. With 740 nm excitation, NADH channel detection is within the 410–490 nm range. With 940 nm excitation, FP channel detection is within the 500–600 nm range. Arrows indicate the transfected cells with respective nuclear FP. Similar to (c), phasor plots correspond to the images to the left, where the top phasor represents the NADH channel that includes all pixels from the whole image. The bottom row is the phasor signal from the segmented nuclei of the transfected cells, shown with the white arrows. The same rainbow LUT was used, and all scale bars represent 10 μm. Intensity values of transfected and untransfected nuclei are plotted on the right of the respective FP. Every dot represents a segmented nucleus. Horizontal bars indicate mean values and vertical lines indicate standard deviations (*$p \leq 0.05$ and **$p \leq 0.001$, from two-tailed t test).

from the emission spectra (Figure 1d). Fluorescence intensity images are color coded using the phasors (false coloring by metabolic arc; see materials and methods) to indicate metabolic ratios of the signal, referred to as phasor-FLIM images (Figure 2b). Phasor plot analysis of the whole images and segmented

nuclei is shown in Figure 2c. Transfected cells can be easily identified using 940 nm excitation and the FP channel (500–600 nm emission). Here, we observe the typical fluorescence of sfGFP, with all pixels mapping close to the phasor unity circle. When excited with 740 nm, fluorescence from the transfected nuclei in the NADH channel (410–490 nm) is distinguishable from untransfected cells. This is indicative of crosstalk and confirms our previous observations that sfGFP excited at 740 nm shows blue-shifted fluorescence similar to recombinant EGFP in solution (Figure S3). This analysis further demonstrates that the phasor coordinates from sfGFP in the NADH channel under 740 nm excitation are located inside the unity circle and thus show unusual properties (short, multi-component lifetimes). This is distinctively different from emission crosstalk when the FP is excited with 940 nm and observed in the NADH channel. In that case, the nuclear pixels map close to the unity circle on the phasor, indicating monoexponential decay, which could easily be unmixed from the NADH fluorescence (Figures 2c and S5). Thus, fluorescence from sfGFP when multiplexed with NADH imaging could be mistaken for a metabolic shift toward glycolysis.

The fluorescence crosstalk from sfGFP might be a particular property of this FP. To investigate if this is a more general property of GFPs, we performed the same experiments with H2B-fused avGFP, mEGFP, and mNG. Phasor-FLIM images and respective phasor plots are shown in Figures 2d–2f. When excited with 940 nm, each FP fluoresces with the expected typical properties (lifetime of 2–3 ns and mapping onto the phasor unity circle). However, when excited with 740 nm, all three green, nuclear-localized FPs fluoresce with a shortened lifetime, and their phasor coordinates overlay with the NADH autofluorescence phasor cloud. This is shown by segmenting nuclei of transfected cells and visualizing the respective phasor plots for these pixels (Figures 2d–2f). It should be noted that the FP-H2B constructs are not homogeneously distributed throughout the nucleus but seem to show a slight preference for nucleoli. Furthermore, we note increased background intensity of nuclei from transfected versus untransfected cells. Quantification of 740 nm excitation-induced nuclear fluorescent intensity over the 410–490 nm emission range shows a statistically significant increase in signal for nuclei expressing avGPF and mEGFP ($p = 0.010$ and 0.001, respectively), indicating significant FP excitation across the NADH detection range. However, while mNG-expressing nuclei show a slight increase in fluorescence signal over untransfected nuclei, the effect appears to not be statistically significant ($p = 0.139$). These results show that while the tested GFPs all share some emission in the NADH range upon 740 nm TPE, the degree of interference with metabolic imaging differs across FPs, therefore indicating that specific GFPs could prove to be better candidates for combined metabolic imaging due to decreased/negligible excitation under NADH imaging conditions. Each of these FPs might impede multiplexed metabolic imaging. Notably, avGFP shows a smaller shift in the phasor cloud when comparing 740 and 940 nm excitation, which could be a result of the blue-shifted fluorescence excitation peak at about 400 nm compared to the other GFPs with excitation peaks around 490 nm (see OPE spectra in Figure S1). As fluorescence lifetime is a function of the local environment of the fluorophore, we confirmed our observations of blue-shifted, short-lifetime fluorescence (mapping toward the center of the phasor plot) in the cellular assay using purified recombinant EGFP in solution (Figure S4A).

For excitation and detection of NADH, various wavelengths have been used in the literature (Table S1). To investigate the effect of TPE wavelengths on fluorescence crosstalk from the FPs, we performed an excitation scan varying excitation wavelengths from 680 to 780 nm and observed fluorescence in the NADH channel (Figure 3). We segmented transfected nuclei, untransfected nuclei, and cytoplasm/mitochondria to compare fluorescence across TPE wavelengths. Transfected nuclei appear with higher mean fluorescence as compared to untransfected controls within the same image. Fluorescence from untransfected nuclei originates from low levels of free NADH in the nuclei. avGFP, mEGFP, and sfGFP show signal levels clearly above background (Figures 3a–3c). Nuclear-localized fluorescence from mNG is slightly lower and closer to the background (Figure 3d), consistent with the cytoplasmic mNG (Figure S2). Given the NADH and FP TPE profiles in the 680–780 nm range, it is difficult to disentangle their respective contributions to total intensity and apparent lifetime. In our hands, 740 nm TPE offers a compromise.

Due to crosstalk between 740 nm-excited GFPs and NADH, we sought to use bathochromic-shifted FPs, expecting to see less excitation crosstalk. We chose three FPs with increasing excitation maxima, mCitrine, stagRFP, and mCherry (516, 555, and 587 nm, respectively), whose OPE excitation and fluorescence spectra are well separated from the NADH channel (Figure S6). We utilized our TPE assay with H2B-FP fusions to experimentally test for crosstalk with metabolic imaging (Figure 4). For all three red-shifted FPs, we observed excitation at 740 nm and fluorescence in the NADH channel. The segmented nuclei of transfected cells are clearly discernible in the

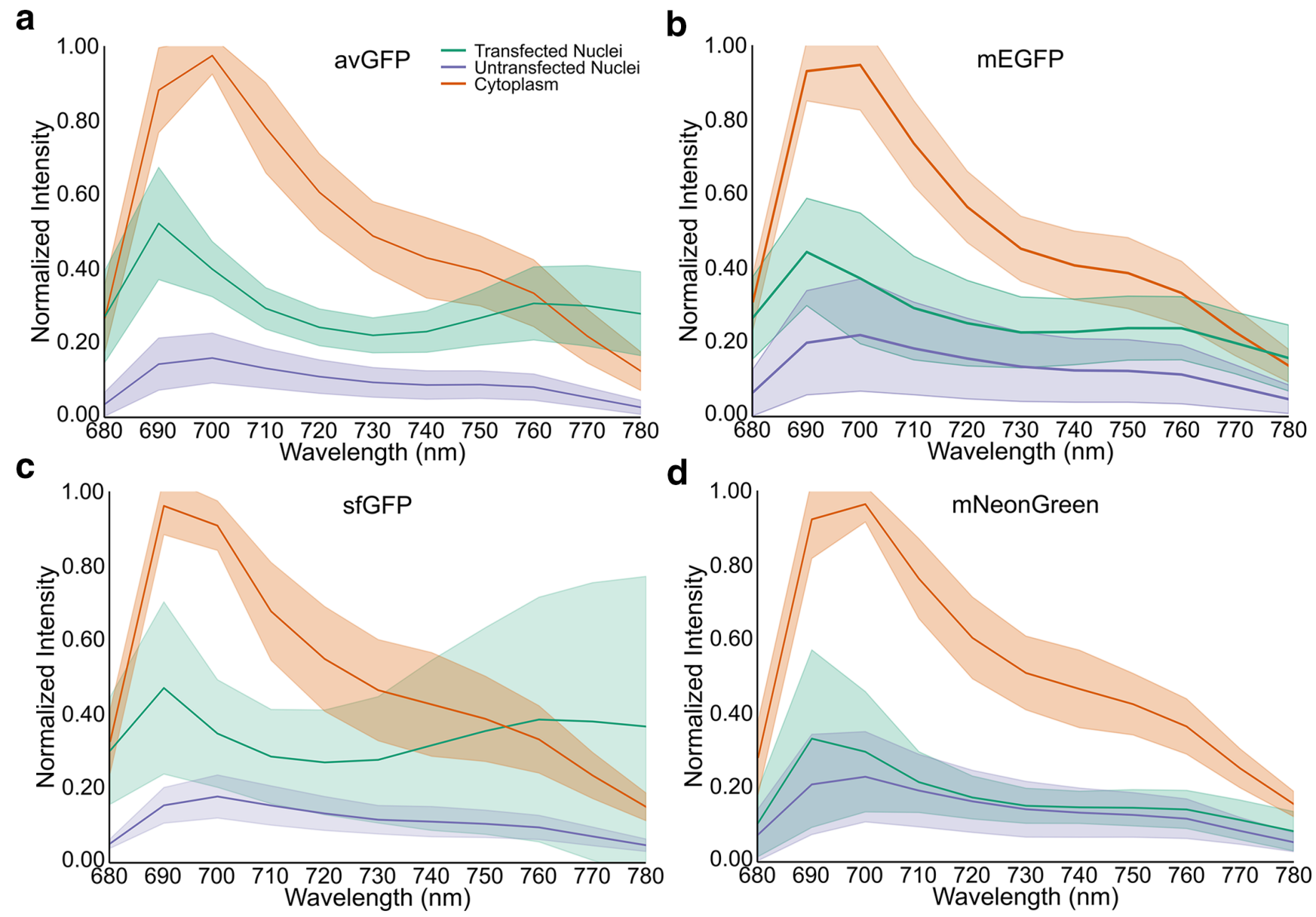

Figure 3. Two-photon excitation scan. Fluorescence intensity measurements in the NADH channel (410–490 nm) from cytoplasm (orange line), untransfected control nuclei (blue line), and transfected nuclei (green line) of U2OS cells across excitation wavelengths from 680–780 nm with 10 nm intervals in between. All measurements were normalized to excitation laser power (see materials and methods). Data points represent mean fluorescence intensities, and shaded regions indicate standard error of the mean. (a) avGFP, (b) EGFP, (c) sfGFP, and (d) mNG. Each data point represents the average of measurements from at least 5 nuclei per condition across multiple biological replicates.

phasor-FLIM images and from the NADH cloud on the phasor plot but might be mistaken for metabolic shifts toward glycolysis. As mentioned above, we performed a TPE scan from 680 to 780 nm and observed above-background fluorescence in the nuclei for all three FPs (Figure 4). mCherry shows the least excitation and fluorescence in the 680–780 nm range as compared to the untransfected background (Figure 4c). We verified the TPE of mCherry at 740 nm and the blue-shifted fluorescence *in vitro* with purified recombinant protein (Figures S4b and S7). Using the recombinant protein, we further showed that the blue-shifted fluorescence is also present using 370 nm excitation (Figure S8). Thus, OPE-based metabolic imaging may suffer from the same crosstalk.

In our hands, shifting the excitation wavelength of the FP did not allow for multiplexing with NADH. Therefore, we tested changing another fluorescence property, the fluorescence lifetime. All thus far tested GFPs have lifetimes on the order of 2–3 ns. We tested short-lifetime monomeric GFP (slmGFP)[39] and bright ultimately short-lifetime enhanced emitter (BrUSLEE)[50] with fluorescence lifetimes of 1.8 and 0.8 ns, respectively, in our TPE crosstalk assay (OPE spectra are shown in Figure S9). At 740 nm, both FPs show weak fluorescence in the NADH channel, mapping close to the NADH cloud on the phasor (Figures 4d and 4e). The position of the signal on the phasor is more similar to that of red-shifted FPs than to other GFPs (shown in Figures 2d–2g). TPE scans (Figures 4a–4c) reveal that fluorescence levels are close to background for excitation < 740 nm, but fluorescence in general (also at 940 nm TPE) is dim.

Recently, a set of monomeric, bright, and photostable FPs based on StayGold (SG)[51] have been developed. Their photobleaching behaviors are distinct from any other FP, implying distinct photophysical properties. We chose mSG2[52] and StayGoldE138D[53] for assessment in our TPE assay, expressing and analyzing H2B fusions as previously. For both SG variants, we observe low TPE at 740 nm, negligible for StayGoldE138D, indicating that fluorescence intensities are comparable to those of untransfected nuclei (Figures 5a and 5b). However, phasor analysis of NADH and FP signals reveals overlap with NADH, similar to other GFPs.

TPE at 740 nm, along with the resulting emission observed in the NADH channel and a shortened lifetime, suggests that the FP chromophore may decay from a specific state distinct from that typically exploited in standard imaging. The chromophores in the GFP-type FPs can be protonated and deprotonated, which can have large effects not only on their absorption but also on their excitation spectra.[34,35,54] Changes in pH can be used to push the chromophore into one or the other state.[37] Alternatively, certain nanobodies have been shown to enhance (E)GFP fluorescence by promoting the

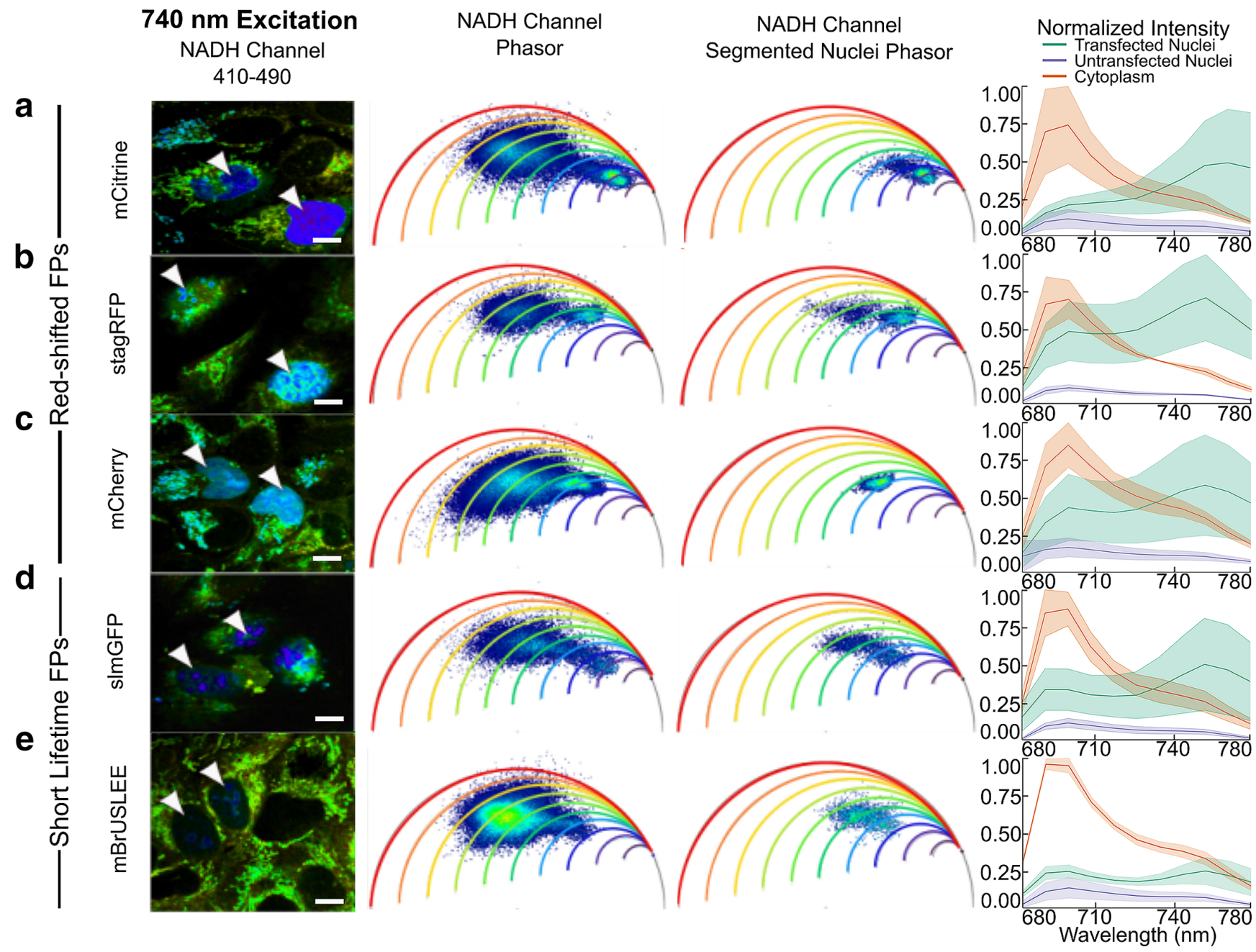

Figure 4. TPE crosstalk assay on bathochromic and short-lifetime FPs. Laser scanning TPE microscopy images of U2OS cells expressing red-shifted nuclear (a) mCitrine, (b) stagRFP, and (c) mCherry and short-lifetime nuclear (d) slmGFP and (e) BrUSLEE with 740 nm excitation. Detection in the NADH channel range 410–490 nm. Arrows indicate the transfected cells with nuclear FPs. Color is based on the phasor plot metabolic arcs with a rainbow LUT to represent the percentage of bound NADH, where blue is 0% and red is 100%. The left phasors represent the NADH channel that includes all pixels from the whole image. The right phasor plots show the signal from the segmented nuclei of the transfected cells, indicated by the white arrows. Scale bars: 10 μm. Fluorescence intensity measurements from cytoplasm (orange line), untransfected control nuclei (blue line), and transfected nuclei (green line) of U2OS cells across excitation wavelengths from 680–780 nm with 10 nm intervals in between. All measurements were normalized to excitation laser power (see materials and methods). Lines represent normalized mean fluorescence intensities, and shaded regions indicate standard error of the mean. Each plot represents the average of measurements from at least 5 nuclei per condition across multiple biological replicates.

anionic (deprotonated) state.[42,55] Building on the notion that a specific fluorescence pathway caused by a distinct chromophore state could cause the short-lifetime fluorescence of the FPs observed in the NADH channel, we co-expressed the enhancer anti-GFP nanobody (vhh) with avGFP-H2B and EGFP-H2B. To ensure equimolar expression, we used the T2A multicistronic system. Figures 5c and 5d show the TPE phasor-FLIM images, phasor plots, and excitation scans. TPE at 740 nm results in above-background fluorescence for avGFP with a phasor position similar to avGFP without vhh (Figure 2f). For vhh-mEGFP, TPE at 740 nm causes minimal fluorescence from transfected nuclei compared with background from untransfected cells. Furthermore, in the presence of vhh, a distinct, longer-lifetime component becomes apparent on the phasor more similar to the phasor position for 940 nm-excited GFPs (Figure 2d). However, some overlap with the metabolic NADH signal remains.

Metabolic signatures can be quite diverse and map all across the phasor plot space. Imaging untransfected cells reveals considerable heterogeneity, even within a single image (Figures 6a and 6b). To map out the expected expansion of the phasor, we used different drug and metabolic treatments to determine the biological minima and maxima for metabolic trajectory analysis in U2OS cells. Control cells, cells at low glucose (LG) and high glucose (HG), and cells treated with rotenone/antimycine A (Rot/AA), an ATP production inhibitor that shuts down OXPHOS through complex I and III, respectively, are shown in Figure 6c. During starvation with LG, cells shift toward OXPHOS. Upon HG stimulation, cells expectedly shift toward glycolysis. With HG + Rot/AA, two separate phasor populations emerge: one corresponding to the expected HG population and the other corresponding to the Rot/AA effect. As Rot/AA is known to inhibit OXPHOS, the cellular metabolism shifts toward glycolysis. Finally, we overlay that with the mean position of the segmented nuclei from transfected cells (Figure 6d). This summary analysis reveals that all tested FPs show a large degree of overlap with the NADH signature. Therefore, the spatial separation of the FP and NADH signals appears as an attractive strategy. We have not observed changes to NADH lifetime in FP-H2B-transfected versus untransfected cells (Figure S10).

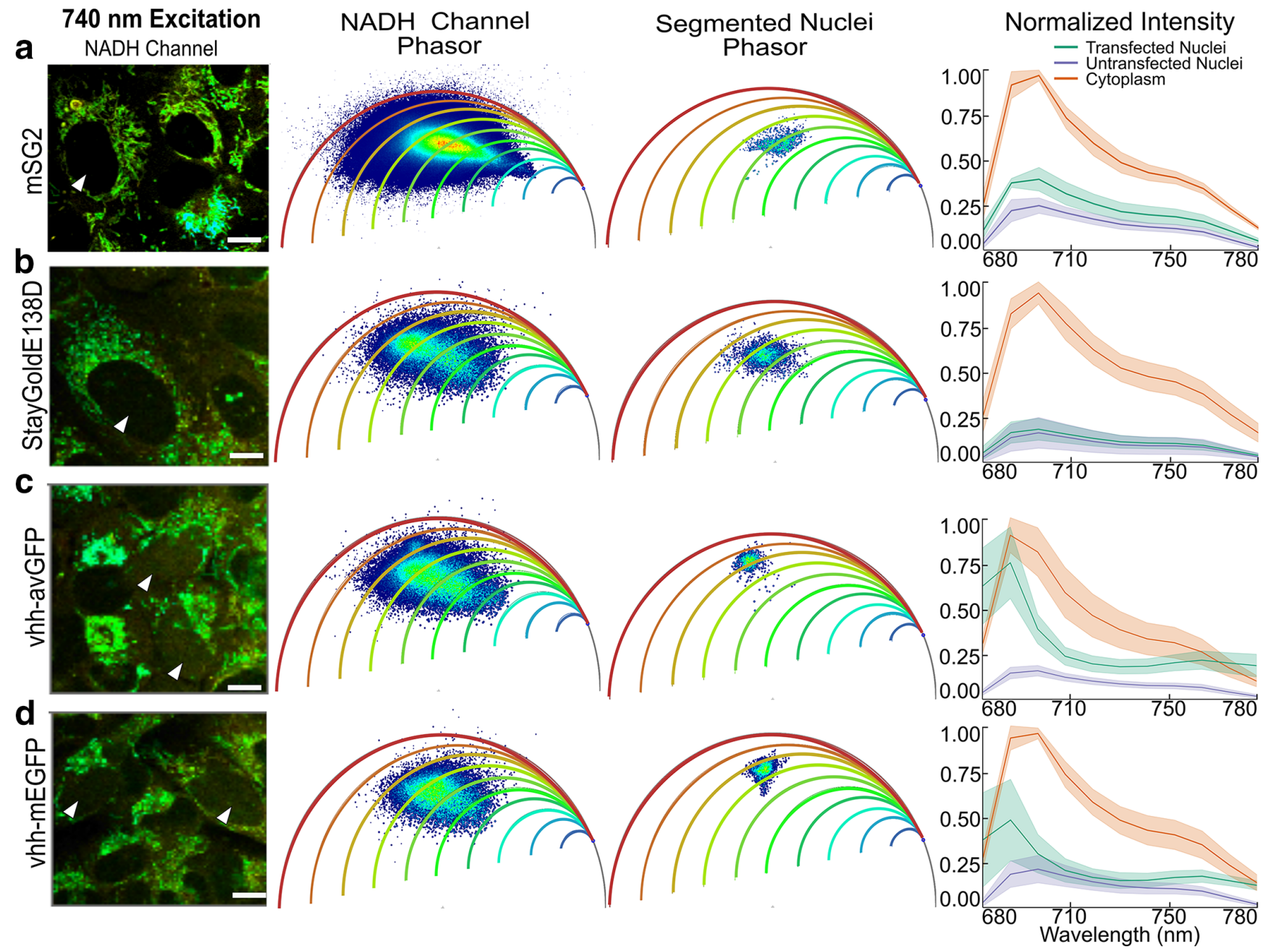

Figure 5. TPE cross-excitation assay using nuclear-localized FPs. Laser scanning TPE microscopy images of U2OS cells expressing nuclear (a) mSG2, (b) StayGoldE138D, (c) vhh-avGFP, and (d) vhh-mEGFP with 740 nm excitation. Detection in the NADH channel range 410–490 nm. Arrows indicate the transfected cells with nuclear FPs. Color is based on the phasor plot metabolic arcs. Phasor plots associated with each FP are shown with metabolic arcs with a rainbow LUT to represent the percentage of bound NADH, where blue is 0% and red is 100%. The left phasor plots represent the NADH channel that includes all pixels from the whole image. The right phasor plots show the signal from the segmented nuclei of the transfected cells, indicated by the white arrows, determined using 940 nm excitation images (data not shown). Scale bars: 10 μm. Fluorescence intensity measurements from cytoplasm (orange line), untransfected control nuclei (blue line), and transfected nuclei (green line) of U2OS cells across excitation wavelengths from 680–780 nm with 10 nm intervals in between. All measurements were normalized to excitation laser power (see materials and methods). Lines represent normalized mean fluorescence intensities, and shaded regions indicate standard error of the mean. Each plot represents the average of measurements from at least 5 nuclei per condition across multiple biological replicates.

DISCUSSION

Metabolic imaging, exploiting TPE of NADH and the change in its fluorescence lifetime when bound to metabolic enzymes, is emerging as a powerful tool for studying metabolism in cells and tissues.[17,21] However, the exact acquisition conditions, for example, NADH TPE wavelength or detection, vary widely across the literature (Table S1). In this study, we try to provide a starting point for optimization; however, exact conditions will depend on the specifics of the biological sample being studied. Our primary focus is on the potential for multiplexing NADH metabolic imaging with other fluorophores, specifically FPs.

Interpreting metabolic imaging data can be challenging and depends on a variety of controls, such as maximum OXPHOS and maximum glycolysis conditions measured for individual samples and studies. Multiplexing NADH FLIM with another label for specific cell types within a tissue or subcellular structures could aid in data interpretation and enable functional studies. Various FPs have been used previously in conjunction with NADH autofluorescence imaging. However, concerns have been raised that GFP and EGFP can be excited at 740 nm, causing a fluorescence signal indistinguishable from that of NADH in metabolic imaging.[29,31,56] These studies have not employed the powerful phasor approach to fluorescence, which has now become a standard tool to visualize metabolic imaging data. Our study combines the use of phasor FLIM and spatial separation of cytoplasmic NADH fluorescence from nuclear-localized FPs. While some NADH fluorescence is expected from the nucleus, it is significantly lower than in the cytoplasm and predominantly comprises free NADH.[57,58] However, we need to acknowledge that the FP signal is likely being mixed with a low level of NADH autofluorescence, and thus in cells, we are quantifying fluorescence changes against this background.

Our experiments using recombinant FPs (Figures S3, S4, and S7) and cytoplasmic sfGFP and mNG (Figures 1 and S2) confirm the reports of blue-shifted fluorescence under 740 nm TPE[29] as well as under OPE (Figure S8). To quantitatively assess the effect and fully harness the power of the phasor transform, we move the FP

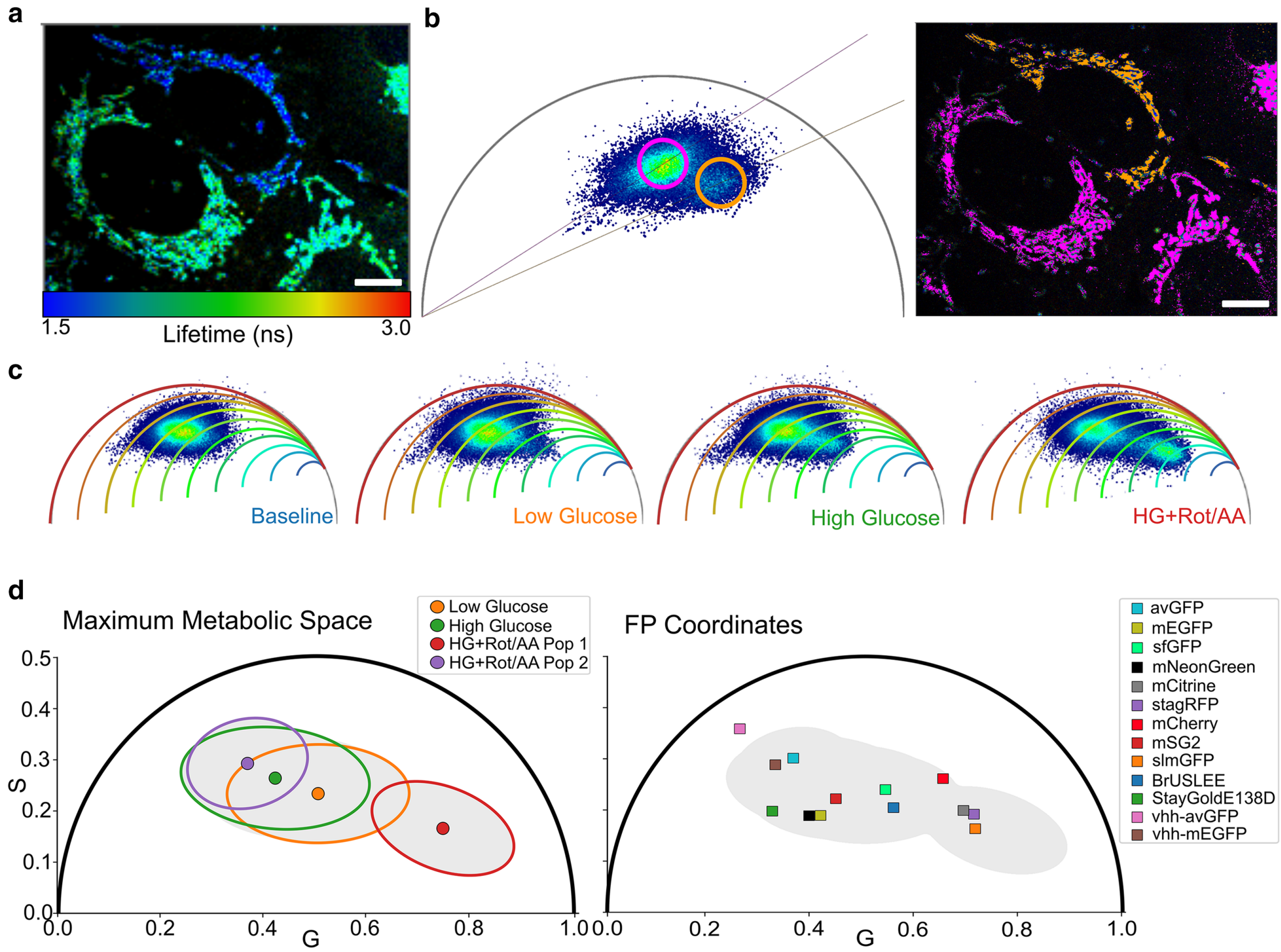

Figure 6. Maximum metabolic phasor and summary of the FP cross-excitation. (a) Laser scanning TPE microscopy image of untransfected U2OS with 740 nm excitation. Detection in the NADH channel range 410–490 nm. Color is based on fast FLIM lifetime (ns) with a rainbow LUT, where blue is the shorter lifetime (1.5 ns) and red is the longer lifetime (3.0 ns). Scale bar: 10 μm. (b) Same image from (a) with color based on the phasor plot selection, where magenta corresponds to the pixels within the magenta circle and orange to the orange circle on the phasor plot to segregate heterogeneous cellular populations with different phasor metabolic signals. (c) Phasors from untransfected U2Os cells from a laser scanning TPE microscopy image with 740 nm excitation and detection in the NADH channel range of 410–490 nm. Phasors are shown with metabolic arcs with a rainbow LUT to represent the percentage of bound NADH, where blue is 0% and red is 100%. The phasor (from left to right) represents cells at baseline (L-15), starved in low glucose (LG), cells stimulated with high glucose (HG), and cells stimulated with HG and introduced to an oxidative phosphorylation inhibitor (HG + Rot/AA). (d) Metabolic trajectory boundary indicated by the gray shaded area, determined by the pixels within two standard deviations of the respective mean G and S coordinates of each condition in (c). LG is orange, HG is green, and HG + Rot/AA is red and purple. The mean G and S coordinate values of FPs are plotted within the metabolic trajectory boundary (right).

signal to the nucleus using H2B fusions. The nuclear physicochemical environment differs from that of the cytoplasm, yet we observe similar fluorescence properties in cyt-mNG/sfGFP compared with H2B-mNG/sfGFP. Strikingly, under NADH imaging conditions (740 nm excitation and 410–490 nm detection), we record increased intensity and a shorter fluorescence lifetime compared with untransfected nuclei in the same sample. The excitation and fluorescence are weak compared to 940 nm TPE; however, because the signal from NADH at 740 nm is also weak, it may still pose an obstacle to effective multiplexing.

We observe excitation at 740 nm and fluorescence within the NADH detection window for several widely used FPs across the visible spectrum. Varying the excitation wavelengths changes the effect slightly, but we could not identify a condition where it is abolished completely. Fluorescence at 740 nm TPE from red-shifted FPs such as mCherry and stagRFP was unexpected and suggests that the observed fluorescence does not originate from crosstalk of the typical fluorescence peak into the NADH channel. Instead, a different state is excited or induced, similar to what has been suggested for dsRed.[31,59] At this point, we cannot exclude that the 740 nm laser induces a photoconversion or color change on the FPs. However, we did not notice an increase in the blue-shifted signal over time. Among the 11 tested FPs, the most promising candidates for multiplexing are mNG, mSG2, and StayGoldE138D. These FPs show negligible excitation at 740 nm and have the benefit of high photostability under OPE.[52,53,60]

The analysis of the TPE spectra in live cells shows that slightly varying the excitation wavelength does

not allow for mitigation of the cross-excitation. A limitation of our assay is that we have no control over the expression levels of the FP-H2B proteins because we were employing transient transfections. Variability in expression explains the large standard deviation in the excitation scans, which are based on the fluorescence intensities of nuclear background, NADH in cytoplasm, and nuclear FPs. Overexpression could also contribute to the inhomogeneous FP distribution in the nuclei.[61] Using stable expression could mitigate that, but we would not expect a change to our observations and conclusions, as fluorescence lifetime is robust against changes in concentration.[62] While we do not observe a change to NADH lifetime for FP-H2B-transfected versus control cells (Figure S10), we cannot entirely exclude that FP expression could cause changes to metabolism, for example, through reactive oxygen species (ROS) generation, during extended illumination.[63–65]

The cause of the undesired fluorescence properties of the FPs under 740 nm TPE remains a matter of further investigation. There is likely a link to the protonated and anionic chromophore forms of GFP-type FPs, as excitation at 800 nm TPE and emission around 460 nm have been reported previously.[34,35,37] Also, other states of the chromophores might contribute, as we observe similar effects for all tested FPs (some of which are evolutionarily distant).[66–68] In our experiments, we are getting some hints from the co-expression of mEGFP and avGFP with the enhancer nanobody (vhh).[42] The vhh is known to shift the FP's chromophore to the deprotonated state, thus forcing a specific fluorescence decay route. This seems to reduce the emission in the NADH channel and shifts it to the FP channel. We co-expressed the nanobody and FP using a T2A system. A tandem version could potentially be more efficient but bears the risk of oligomerization.

Our findings implicate challenges to multiplex NADH with FPs as reporters. We aim to raise awareness that cross-excitation represents a widespread phenomenon, influencing FPs across the visible spectrum rather than merely green variants. FP emission spectra under NADH imaging conditions (e.g., 740 nm TPE) can differ from those obtained at their conventional excitation maxima, indicating that standard emission spectra alone may not predict the magnitude of blue-shifted fluorescence under NADH excitation. While our study focuses on TPE metabolic imaging, OPE metabolic imaging with excitation of NADH < 375 nm has been reported.[69,70] Also, under UV excitation, we observe signals in the NADH channel (~460 nm; Figure S10). We strongly recommend the spatial segregation of FP signals from endogenous NADH; for instance, directing FPs to the membrane or the nucleus (as shown here with H2B fusions) effectively isolating the two signals. Careful analysis and controls are paramount: evaluating untransfected and transfected cells within the same field of view allows for the identification and possibly the subtraction of crosstalk contributions. While stable, uniform FP expression may permit the use of constant background corrections to yield reliable metabolic data, variable expression, prevalent in transient transfections or rapidly developing tissue, introduces cell-to-cell intensity fluctuations that impede measurements of metabolic signatures, potentially inducing systematic errors. Consequently, multiplexed strategies in these contexts require extreme caution. Among the FPs evaluated in this work, StayGoldE138D and mNG are identified as the most compatible candidates; both demonstrate negligible excitation in the NADH channel under 740 nm TPE and are thus preferable to standard alternatives such as mCherry.

Overall, we describe the observation that multiplexing metabolic imaging of NADH at 740 nm TPE with FPs is challenging. This is a cautionary tale that provides some insights into the underlying mechanisms and issues. We provide a path toward a solution and remain cautiously optimistic that with the right controls and spatial separation of the signals, multiplexing of NADH and FPs will be possible.

DATA AND CODE AVAILABILITY

- All code used to analyze data is available at https://github.com/janiellecuala/Multiplexing-NADH-and-Fluorescent-Proteins-in-Metabolic-Imaging.
- All raw data can be requested from the corresponding author upon reasonable request.

ACKNOWLEDGMENTS

F.S. acknowledges support from the long-term postdoctoral fellowships from EMBO (EMBO ALTF 849-2020) and HFSP (LT000404/2021-L) as well as from the Leverhulme Trust, UK (LIP-2021-017). O.A.d.l.F. acknowledges support from the USC NIH T32 PhD Fellowship in Developmental Biology, Stem Cells, and Regeneration (5T32HD060549). J.C. acknowledges support from the ARCS Foundation and CHLA TSRI Core Pilot Grant. L.C. acknowledges support and guidance from Stacey D. Finley. The authors thank Tristan Nicholas (University of Warwick) for critical proofreading of the manuscript and Jason Junge and Arkadi Shwartz at the Translational Imaging Center (USC) for technical support and maintenance of the Leica SP8.

AUTHOR CONTRIBUTIONS

Conceptualization, F.S.; methodology, F.S., J.C., and O.A.d.l.F.; software, F.S. and J.C.; formal analysis, J.C., O.A.d.l.F., and L.C.; investigation, F.S., J.C., O.A.d.l.F., Y.P., and L.C.; resources, O.A.d.l.F., Y.P., and L.C.; data curation, T.S., C.D., and A.V.; visualization,

J.C., O.A.d.l.F., and L.C.; writing – original draft, F.S.; writing – review & editing, F.S., J.C., O.A.d.l.F., L.C., K.W., S.E.F., S.K.G., and S.A.K.; supervision, F.S., K.W., S.E.F., S.K.G., and S.A.K.; funding acquisition, K.W., S.E.F., S.K.G., and S.A.K.

DECLARATION OF INTERESTS

The authors declare no competing interests.

SUPPLEMENTAL INFORMATION

Supplemental information can be found online at https://doi.org/10.1016/j.bpr.2026.100294.

REFERENCES

1. DeBerardinis, R. J., and C. B. Thompson. 2012. Cellular metabolism and disease: what do metabolic outliers teach us? *Cell.* 148:1132–1144. https://doi.org/10.1016/j.cell.2012.02.032.
2. Hu, W., and M. A. Lazar. 2022. Modelling metabolic diseases and drug response using stem cells and organoids. *Nat. Rev. Endocrinol.* 18:744–759. https://doi.org/10.1038/s41574-022-00733-z.
3. Romero-Garcia, S., J. S. Lopez-Gonzalez, …, H. Prado-Garcia. 2011. Tumor cell metabolism. *Cancer Biol. Ther.* 12:939–948. https://doi.org/10.4161/cbt.12.11.18140.
4. Martínez-Reyes, I., and N. S. Chandel. 2021. Cancer metabolism: looking forward. *Nat. Rev. Cancer.* 21:669–680. https://doi.org/10.1038/s41568-021-00378-6.
5. Codo, A. C., G. G. Davanzo, …, P. M. Moraes-Vieira. 2020. Elevated Glucose Levels Favor SARS-CoV-2 Infection and Monocyte Response through a HIF-1α/Glycolysis-Dependent Axis. *Cell Metab.* 32:437–446.e5. https://doi.org/10.1016/j.cmet.2020.07.007.
6. Haythorne, E., M. Rohm, …, F. M. Ashcroft. 2019. Diabetes causes marked inhibition of mitochondrial metabolism in pancreatic β-cells. *Nat. Commun.* 10:2474. https://doi.org/10.1038/s41467-019-10189-x.
7. Zhang, J., E. Nuebel, …, M. A. Teitell. 2012. Measuring energy metabolism in cultured cells, including human pluripotent stem cells and differentiated cells. *Nat. Protoc.* 7:1068–1085. https://doi.org/10.1038/nprot.2012.048.
8. Younger, A. K. D., N. C. Dalvie, …, J. N. Leonard. 2017. Engineering Modular Biosensors to Confer Metabolite-Responsive Regulation of Transcription. *ACS Synth. Biol.* 6:311–325. https://doi.org/10.1021/acssynbio.6b00184.
9. Choe, M., and D. V. Titov. 2022. Genetically encoded tools for measuring and manipulating metabolism. *Nat. Chem. Biol.* 18:451–460. https://doi.org/10.1038/s41589-022-01012-8.
10. Tantama, M., J. R. Martínez-François, …, G. Yellen. 2013. Imaging energy status in live cells with a fluorescent biosensor of the intracellular ATP-to-ADP ratio. *Nat. Commun.* 4:2550. https://doi.org/10.1038/ncomms3550.
11. Du, Y., H. Hu, …, T. Wei. 2019. Genetically Encoded FapR-NLuc as a Biosensor to Determine Malonyl-CoA in Situ at Subcellular Scales. *Bioconjug. Chem.* 30:826–832. https://doi.org/10.1021/acs.bioconjchem.8b00920.
12. Ellis, J. M., and M. J. Wolfgang. 2012. A Genetically Encoded Metabolite Sensor for Malonyl-CoA. *Chem Biol.* 19:1333–1339. https://doi.org/10.1016/j.chembiol.2012.08.018.
13. Botman, D., A. Tielman, …, B. Teusink. 2026. An mTurquoise2-Based Glucose Biosensor. *ACS Sens.* 11:1228–1238. https://doi.org/10.1021/acssensors.5c03325.
14. Hung, Y. P., J. G. Albeck, …, G. Yellen. 2011. Imaging cytosolic NADH-NAD+ redox state with a genetically encoded fluorescent biosensor. *Cell Metab.* 14:545–554. https://doi.org/10.1016/j.cmet.2011.08.012.
15. Gest, A. M. M., A. Z. Sahan, …, J. Zhang. 2024. Molecular Spies in Action: Genetically Encoded Fluorescent Biosensors Light up Cellular Signals. *Chem. Rev.* 124:12573–12660. https://doi.org/10.1021/acs.chemrev.4c00293.
16. Koveal, D., C. M. Díaz-García, and G. Yellen. 2020. Fluorescent Biosensors for Neuronal Metabolism and the Challenges of Quantitation. *Curr. Opin. Neurobiol.* 63:111–121. https://doi.org/10.1016/j.conb.2020.02.011.
17. Luu, P., S. E. Fraser, and F. Schneider. 2024. More than double the fun with two-photon excitation microscopy. *Commun. Biol.* 7:364. https://doi.org/10.1038/s42003-024-06057-0.
18. Lakowicz, J. R., H. Szmacinski, …, M. L. Johnson. 1992. Fluorescence lifetime imaging of free and protein-bound NADH. *Proc. Natl. Acad. Sci.* 89:1271–1275. https://doi.org/10.1073/pnas.89.4.1271.
19. Skala, M. C., K. M. Riching, …, N. Ramanujam. 2007. In vivo multiphoton microscopy of NADH and FAD redox states, fluorescence lifetimes, and cellular morphology in precancerous epithelia. *Proc. Natl. Acad. Sci. USA.* 104:19494–19499. https://doi.org/10.1073/pnas.0708425104.
20. Sharick, J. T., P. F. Favreau, …, M. C. Skala. 2018. Protein-bound NAD(P)H Lifetime is Sensitive to Multiple Fates of Glucose Carbon. *Sci. Rep.* 8:5456. https://doi.org/10.1038/s41598-018-23691-x.
21. Stringari, C., A. Cinquin, …, E. Gratton. 2011. Phasor approach to fluorescence lifetime microscopy distinguishes different metabolic states of germ cells in a live tissue. *Proc. Natl. Acad. Sci. USA.* 108:13582–13587. https://doi.org/10.1073/pnas.1108161108.
22. Digman, M. A., V. R. Caiolfa, …, E. Gratton. 2008. The phasor approach to fluorescence lifetime imaging analysis. *Biophys. J.* 94:L14–L16. https://doi.org/10.1529/biophysj.107.120154.
23. Ranjit, S., L. Malacrida, …, E. Gratton. 2018. Fit-free analysis of fluorescence lifetime imaging data using the phasor approach. *Nat. Protoc.* 13:1979–2004. https://doi.org/10.1038/s41596-018-0026-5.
24. Ranjit, S., R. Datta, …, E. Gratton. 2019. Multicomponent Analysis of Phasor Plot in a Single Pixel to Calculate Changes of Metabolic Trajectory in Biological Systems. *J. Phys. Chem. A.* 123:9865–9873. https://doi.org/10.1021/acs.jpca.9b07880.
25. Stringari, C., J. L. Nourse, …, E. Gratton. 2012. Phasor Fluorescence Lifetime Microscopy of Free and Protein-Bound NADH Reveals Neural Stem Cell Differentiation Potential. *PLoS One.* 7:e48014. https://doi.org/10.1371/journal.pone.0048014.
26. Sameni, S., A. Syed, …, M. A. Digman. 2016. The phasor-FLIM fingerprints reveal shifts from OXPHOS to enhanced glycolysis in Huntington Disease. *Sci. Rep.* 6:34755. https://doi.org/10.1038/srep34755.
27. Snyder, G. A., S. Kumar, …, K. Ray. 2023. Two-photon fluorescence lifetime imaging microscopy of NADH metabolism in HIV-1 infected cells and tissues. *Front. Immunol.* 14:1213180. https://doi.org/10.3389/fimmu.2023.1213180.
28. Paillon, N., T. P. L. Ung, …, C. Hivroz. 2024. Label-free single-cell live imaging reveals fast metabolic switch in T lymphocytes. *Mol. Biol. Cell.* 35:ar11. https://doi.org/10.1091/mbc.E23-01-0009.
29. York, E. M., N. L. Weilinger, …, B. A. MacVicar. 2019. Green fluorescent protein emission obscures metabolic fluorescent lifetime imaging of NAD(P)H. *Biomed. Opt. Express.* 10:4381–4394. https://doi.org/10.1364/BOE.10.004381.
30. Jung, G., J. Wiehler, and A. Zumbusch. 2005. The Photophysics of Green Fluorescent Protein: Influence of the Key Amino Acids

at Positions 65, 203, and 222. *Biophys. J.* 88:1932–1947. https://doi.org/10.1529/biophysj.104.044412.

31. Hess, S. T., E. D. Sheets, …, A. A. Heikal. 2003. Quantitative Analysis of the Fluorescence Properties of Intrinsically Fluorescent Proteins in Living Cells. *Biophys. J.* 85:2566–2580. https://doi.org/10.1016/S0006-3495(03)74679-7.
32. Volkmer, A., V. Subramaniam, …, T. M. Jovin. 2000. One- and Two-Photon Excited Fluorescence Lifetimes and Anisotropy Decays of Green Fluorescent Proteins. *Biophys. J.* 78:1589–1598. https://doi.org/10.1016/S0006-3495(00)76711-7.
33. Costantini, L. M., M. Baloban, …, E. L. Snapp. 2015. A palette of fluorescent proteins optimized for diverse cellular environments. *Nat. Commun.* 6:7670. https://doi.org/10.1038/ncomms8670.
34. Chattoraj, M., B. A. King, …, S. G. Boxer. 1996. Ultra-fast excited state dynamics in green fluorescent protein: multiple states and proton transfer. *Proc. Natl. Acad. Sci. USA.* 93:8362–8367. https://doi.org/10.1073/pnas.93.16.8362.
35. Volkmer, A., V. Subramaniam, …, T. M. Jovin. 2000. One- and two-photon excited fluorescence lifetimes and anisotropy decays of green fluorescent proteins. *Biophys. J.* 78:1589–1598. https://doi.org/10.1016/S0006-3495(00)76711-7.
36. Jung, G., J. Wiehler, and A. Zumbusch. 2005. The photophysics of green fluorescent protein: influence of the key amino acids at positions 65, 203, and 222. *Biophys. J.* 88:1932–1947. https://doi.org/10.1529/biophysj.104.044412.
37. Heikal, A. A., S. T. Hess, and W. W. Webb. 2001. Multiphoton molecular spectroscopy and excited-state dynamics of enhanced green fluorescent protein (EGFP): acid–base specificity. *Chem. Phys.* 274:37–55. https://doi.org/10.1016/s0301-0104(01)00486-4.
38. Salomonnson, E., L. A. Mihalko, …, G. D. Luker. 2012. Cell-based and in vivo spectral analysis of fluorescent proteins for multiphoton microscopy. *J. Biomed. Opt.* 17:096001. https://doi.org/10.1117/1.JBO.17.9.096001.
39. Štefl, M., K. Herbst, …, M. Knop. 2020. Single-Color Fluorescence Lifetime Cross-Correlation Spectroscopy In Vivo. *Biophys. J.* 119:1359–1370. https://doi.org/10.1016/j.bpj.2020.06.039.
40. Lambert, T. J. 2019. FPbase: a community-editable fluorescent protein database. *Nat. Methods.* 16:277–278. https://doi.org/10.1038/s41592-019-0352-8.
41. Farrants, H., M. Tarnawski, …, K. Johnsson. 2020. Chemogenetic Control of Nanobodies. *Nat. Methods.* 17:279–282. https://doi.org/10.1038/s41592-020-0746-7.
42. Kirchhofer, A., J. Helma, …, U. Rothbauer. 2010. Modulation of protein properties in living cells using nanobodies. *Nat. Struct. Mol. Biol.* 17:133–138. https://doi.org/10.1038/nsmb.1727.
43. Goedhart, J., L. van Weeren, …, T. W. J. Gadella, Jr. 2011. Quantitative Co-Expression of Proteins at the Single Cell Level – Application to a Multimeric FRET Sensor. *PLoS One.* 6:e27321. https://doi.org/10.1371/journal.pone.0027321.
44. Schindelin, J., I. Arganda-Carreras, …, A. Cardona. 2012. Fiji: an open-source platform for biological-image analysis. *Nat. Methods.* 9:676–682. https://doi.org/10.1038/nmeth.2019.
45. Cranfill, P. J., B. R. Sell, …, D. W. Piston. 2016. Quantitative assessment of fluorescent proteins. *Nat. Methods.* 13:557–562. https://doi.org/10.1038/nmeth.3891.
46. Zhu, S., D. Larkin, …, P. Arvan. 2016. Monitoring C-Peptide Storage and Secretion in Islet β-Cells In Vitro and In Vivo. *Diabetes.* 65:699–709. https://doi.org/10.2337/db15-1264.
47. Ferri, G., M. Tesi, …, F. Cardarelli. 2020. Metabolic response of Insulinoma 1E cells to glucose stimulation studied by fluorescence lifetime imaging. *FASEB Bioadv.* 2:409–418. https://doi.org/10.1096/fba.2020-00014.
48. Zhao, Y., Y. Yang, and J. Loscalzo. 2014. Real time Measurement of Metabolic States in Living Cells using Genetically-encoded NADH Sensors. *Methods Enzymol.* 542:349–367.
49. Zhang, Q., D. W. Piston, and R. H. Goodman. 2002. Regulation of Corepressor Function by Nuclear NADH. *Science.* 295:1895–1897. https://doi.org/10.1126/science.1069300.
50. Mamontova, A. V., I. D. Solovyev, …, A. M. Bogdanov. 2018. Bright GFP with subnanosecond fluorescence lifetime. *Sci. Rep.* 8:13224. https://doi.org/10.1038/s41598-018-31687-w.
51. Hirano, M., R. Ando, …, A. Miyawaki. 2022. A highly photostable and bright green fluorescent protein. *Nat. Biotechnol.* 40:1132–1142. https://doi.org/10.1038/s41587-022-01278-2.
52. Ando, R., S. Shimozono, …, A. Miyawaki. 2024. StayGold variants for molecular fusion and membrane-targeting applications. *Nat. Methods.* 21:648–656. https://doi.org/10.1038/s41592-023-02085-6.
53. Ivorra-Molla, E., D. Akhuli, …, M. K. Balasubramanian. 2024. A monomeric StayGold fluorescent protein. *Nat. Biotechnol.* 42:1368–1371. https://doi.org/10.1038/s41587-023-02018-w.
54. Kneen, M., J. Farinas, …, A. S. Verkman. 1998. Green Fluorescent Protein as a Noninvasive Intracellular pH Indicator. *Biophys. J.* 74:1591–1599. https://doi.org/10.1016/S0006-3495(98)77870-1.
55. Schneider, F., T. Sych, …, E. Sezgin. 2021. Influence of nanobody binding on fluorescence emission, mobility, and organization of GFP-tagged proteins. *iScience.* 24:101891. https://doi.org/10.1016/j.isci.2020.101891.
56. Bernier, L.-P., E. M. York, …, B. A. MacVicar. 2020. Microglial metabolic flexibility supports immune surveillance of the brain parenchyma. *Nat. Commun.* 11:1559. https://doi.org/10.1038/s41467-020-15267-z.
57. Blacker, T. S., Z. F. Mann, …, M. R. Duchen. 2014. Separating NADH and NADPH fluorescence in live cells and tissues using FLIM. *Nat. Commun.* 5:3936. https://doi.org/10.1038/ncomms4936.
58. Wright, B. K., L. M. Andrews, …, E. Gratton. 2012. NADH Distribution in Live Progenitor Stem Cells by Phasor-Fluorescence Lifetime Image Microscopy. *Biophys. J.* 103:L7–L9. https://doi.org/10.1016/j.bpj.2012.05.038.
59. Marchant, J. S., G. E. Stutzmann, …, I. Parker. 2001. Multiphoton-evoked color change of DsRed as an optical highlighter for cellular and subcellular labeling. *Nat. Biotechnol.* 19:645–649. https://doi.org/10.1038/90249.
60. Shaner, N. C., G. G. Lambert, …, J. Wang. 2013. A bright monomeric green fluorescent protein derived from Branchiostoma lanceolatum. *Nat. Methods.* 10:407–409. https://doi.org/10.1038/nmeth.2413.
61. Musinova, Y. R., O. M. Lisitsyna, …, E. V. Sheval. 2011. Nucleolar localization/retention signal is responsible for transient accumulation of histone H2B in the nucleolus through electrostatic interactions. *Biochim. Biophys. Acta.* 1813:27–38. https://doi.org/10.1016/j.bbamcr.2010.11.003.
62. Berezin, M. Y., and S. Achilefu. 2010. Fluorescence Lifetime Measurements and Biological Imaging. *Chem. Rev.* 110:2641–2684. https://doi.org/10.1021/cr900343z.
63. Onukwufor, J. O., A. J. Trewin, …, A. P. Wojtovich. 2020. Quantification of reactive oxygen species production by the red fluorescent proteins KillerRed, SuperNova and mCherry. *Free Radic. Biol. Med.* 147:1–7. https://doi.org/10.1016/j.freeradbiomed.2019.12.008.
64. Ansari, A. M., A. K. Ahmed, …, Z. Sun. 2016. Cellular GFP Toxicity and Immunogenicity: Potential Confounders in in Vivo Cell Tracking Experiments. *Stem Cell Rev.* 12:553–559. https://doi.org/10.1007/s12015-016-9670-8.
65. Shen, Y., Y. Chen, …, R. E. Campbell. 2017. Engineering of mCherry variants with long Stokes shift, red-shifted

fluorescence, and low cytotoxicity. *PLoS One.* 12:e0171257. https://doi.org/10.1371/journal.pone.0171257.

66. Ghosh, A., S. Isbaner, …, N. Karedla. 2017. Quantifying Microsecond Transition Times Using Fluorescence Lifetime Correlation Spectroscopy. *J. Phys. Chem. Lett.* 8:6022–6028. https://doi.org/10.1021/acs.jpclett.7b02707.

67. Meech, S. R. 2009. Excited state reactions in fluorescent proteins. *Chem. Soc. Rev.* 38:2922–2934. https://doi.org/10.1039/b820168b.

68. Steiert, F., E. P. Petrov, …, T. Weidemann. 2018. Photophysical Behavior of mNeonGreen, an Evolutionarily Distant Green Fluorescent Protein. *Biophys. J.* 114:2419–2431. https://doi.org/10.1016/j.bpj.2018.04.013.

69. Schneckenburger, H., M. Wagner, …, R. Sailer. 2004. Autofluorescence lifetime imaging of cultivated cells using a UV picosecond laser diode. *J. Fluoresc.* 14:649–654. https://doi.org/10.1023/b:jofl.0000039351.09916.cc.

70. Samimi, K., D. E. Desa, …, M. C. Skala. 2023. Light-sheet autofluorescence lifetime imaging with a single-photon avalanche diode array. *J. Biomed. Opt.* 28:066502. https://doi.org/10.1117/1.JBO.28.6.066502.

Biophysical Reports, Volume *6*

Supplemental information

Bright probes, blurred metabolism: Navigating fluorescent protein cross-excitation in NADH FLIM

Janielle Cuala, Oscar Alberto de la Fuente, Lynne Cherchia, Yuanzhong Pan, Tvisha Singh, Chris Deng, Andrea Velazquez, Kate White, Senta K. Georgia, Steve A. Kay, Scott E. Fraser, and Falk Schneider

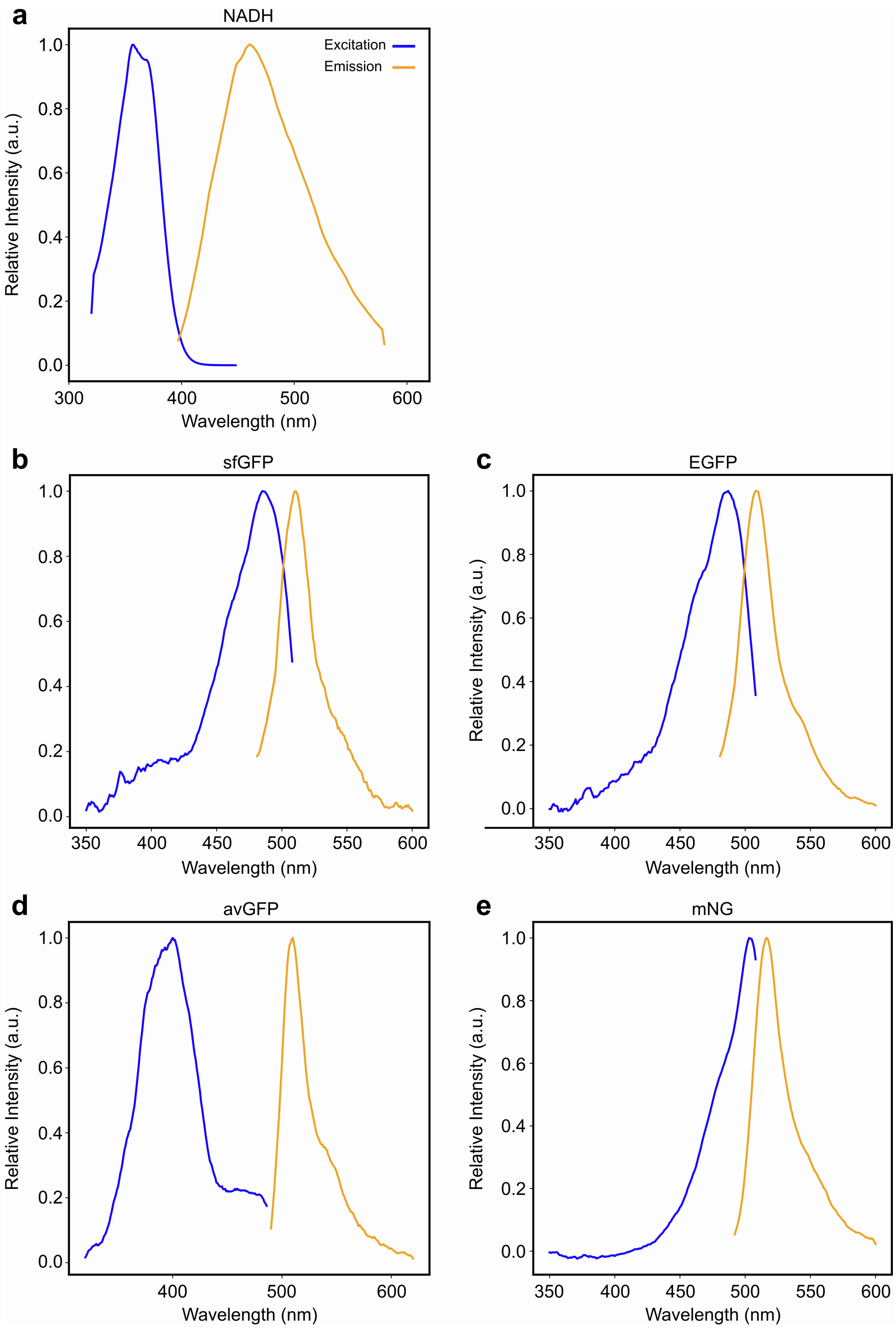

Figure S1: One photon excitation and emission spectra of NADH in solution and green fluorescent proteins expressed in U2OS cells as H2B fusions. Blue curves are the excitation spectra, and the yellow curves are the emission spectra.
a NADH in water
b sfGFP in cells
c mEGFP

d avGFP
e mNG
Spectra were acquired in glass bottom 96 well plates on a fluorescence plate reader. Lines represent averages of multiple repeats.

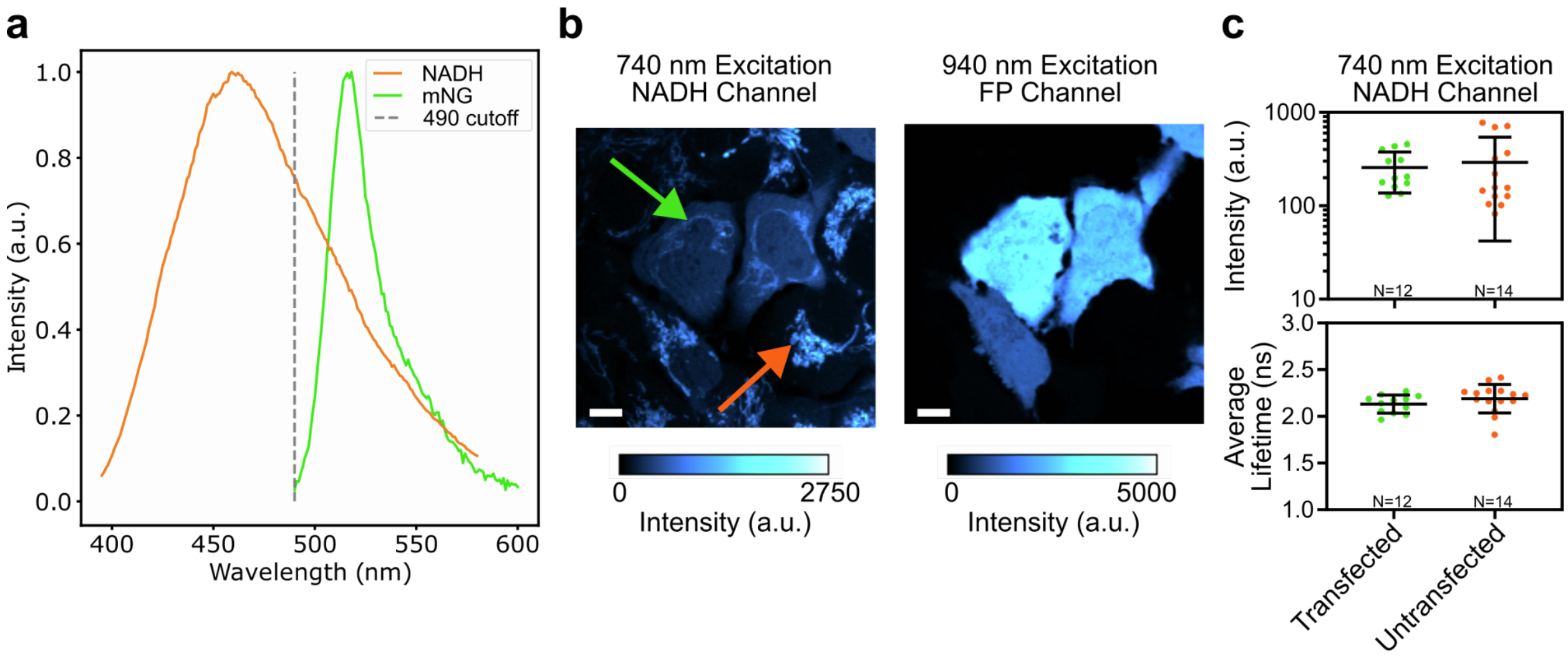

Figure S2: Cross-talk of NADH and mNeonGreen (mNG).

a Spectral overlap of NADH (in water) and mNG-H2B expressed in HEK cells

b Laser scanning TPE microscopy images of cells expressing cytoplasmic mNG. Left: Excitation with 740 nm. Right: Excitation with 940 nm. Arrows indicate mitochondria in transfected (green) and untransfected (orange) cells. Scale bars are 10 µm.

c Fluorescence intensity and lifetime of segmented cytoplasm in transfected (green) and untransfected (orange) cells. Every dot represents one cell. Horizontal bar is mean value and vertical bars standard deviation.

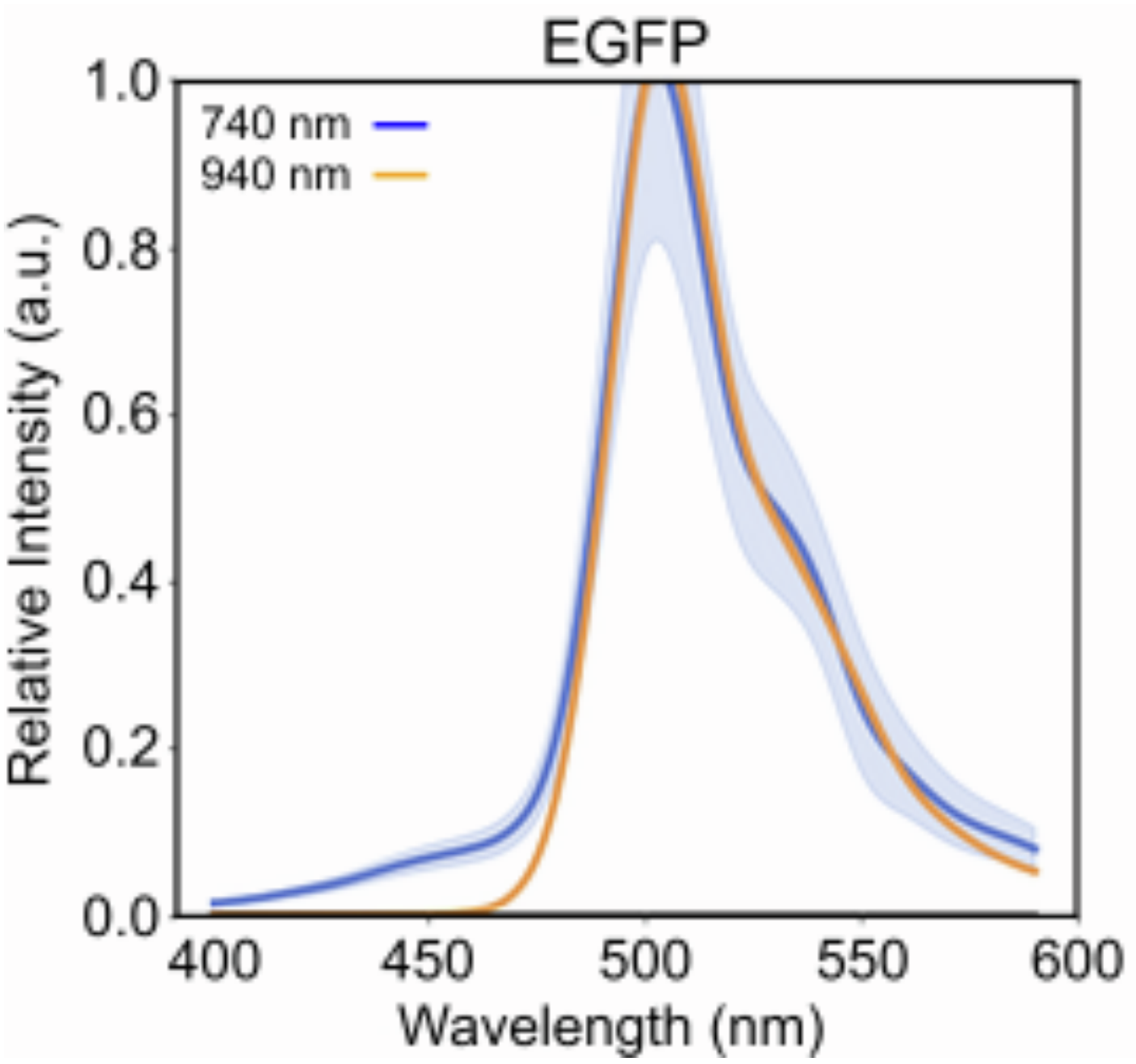

Figure S3: Two-photon emission spectra of recombinant EGFP in L-15.
150 nM solution of EGFP in L-15 was excited using 740 nm (blue) and 940 nm (orange). Values are averaged across an image and the shaded area represents standard deviation.

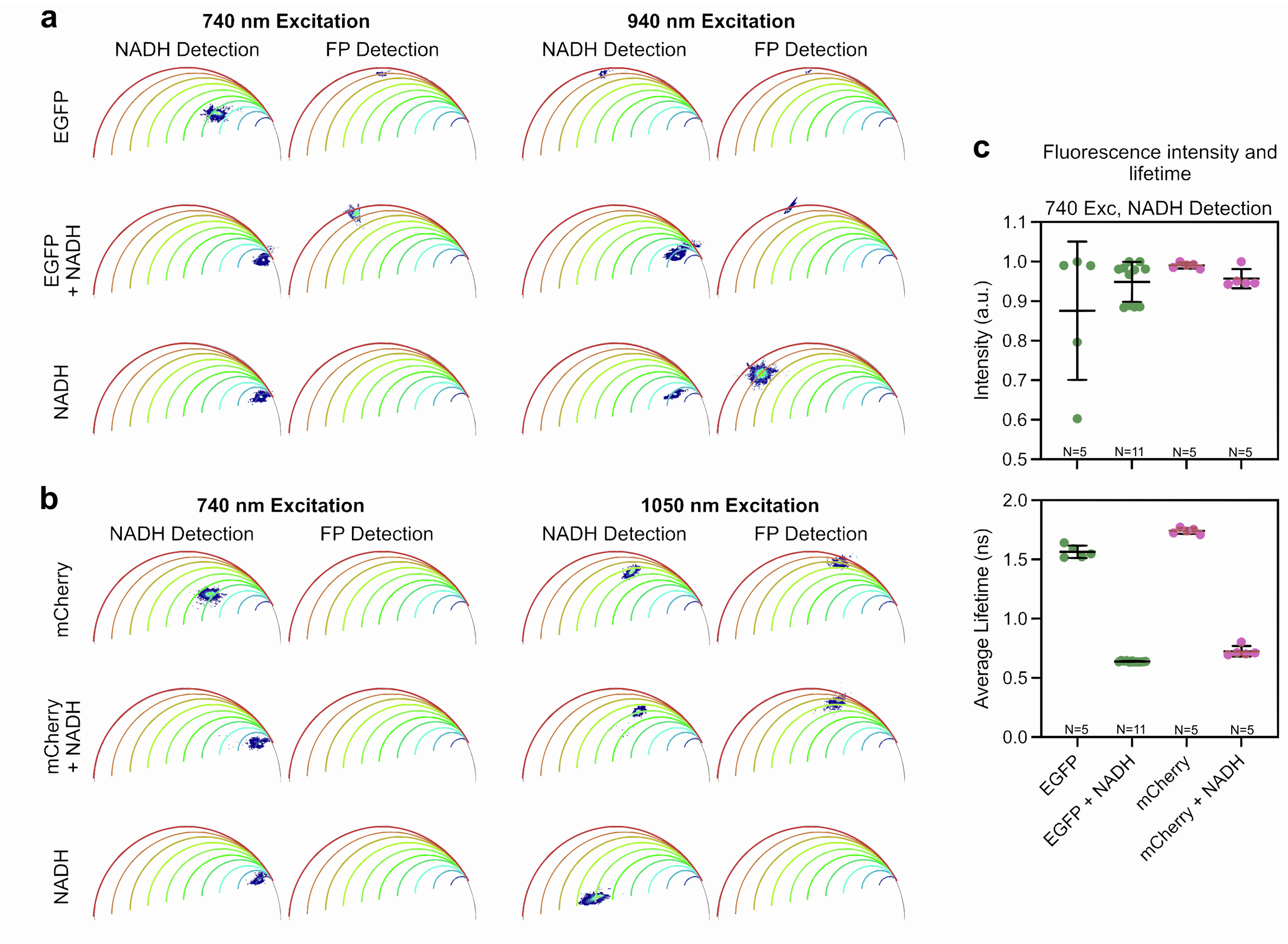

Figure S4: Excitation of purified recombinant EGFP and mCherry in the presence and absence of NADH in L-15. EGFP is imaged at a concentration of 150 nM, mCherry is imaged at 250 nM, and NADH is imaged at 200 μM.

a Phasors for recombinant EGFP and NADH fluorescence lifetime. Left: Excitation with 740 nm and NADH detection (410-490 nm, left) and FP detection (500-600 nm, right). Right: Excitation with 940 nm and NADH detection (left) and FP detection (right).

b Phasors for recombinant mCherry and NADH fluorescence lifetime. Left: Excitation with 740 nm and NADH detection (410-490 nm, left) and FP detection (600-700 nm, right). Right: Excitation with 1050 nm and NADH detection (left) and FP detection (right).

c Fluorescence lifetime and intensity of recombinant FPs with 740 nm excitation and NADH detection. Horizontal bars indicate mean value and vertical bars indicate standard deviation.

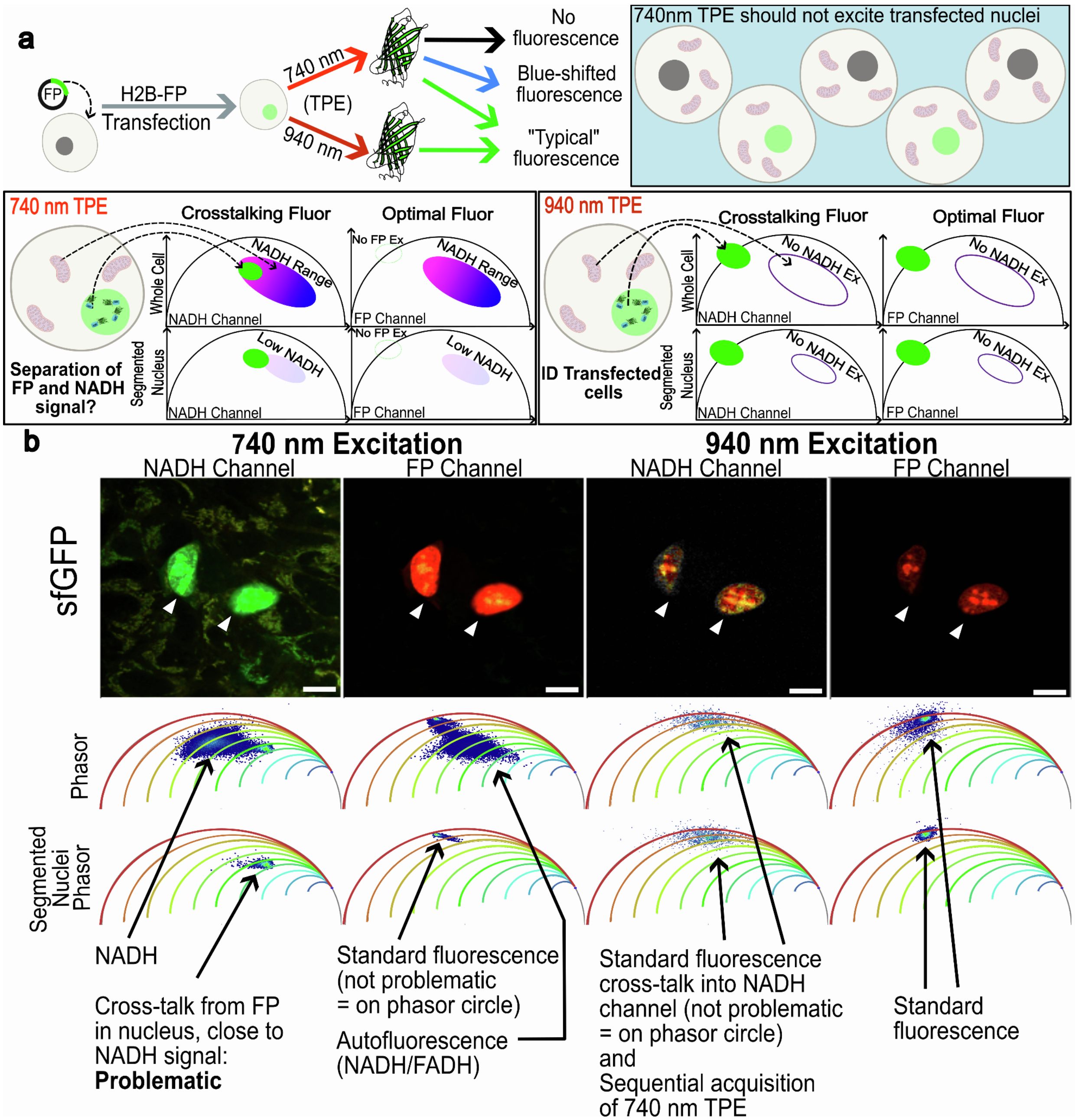

Figure S5: Cross-excitation assay.

a Extended cartoon explaining the cross-excitation assay (see also Fig. 2a).

b Cross-excitation assay of sfGFP same as in Fig. 2b,c with added annotations to aid data interpretation.

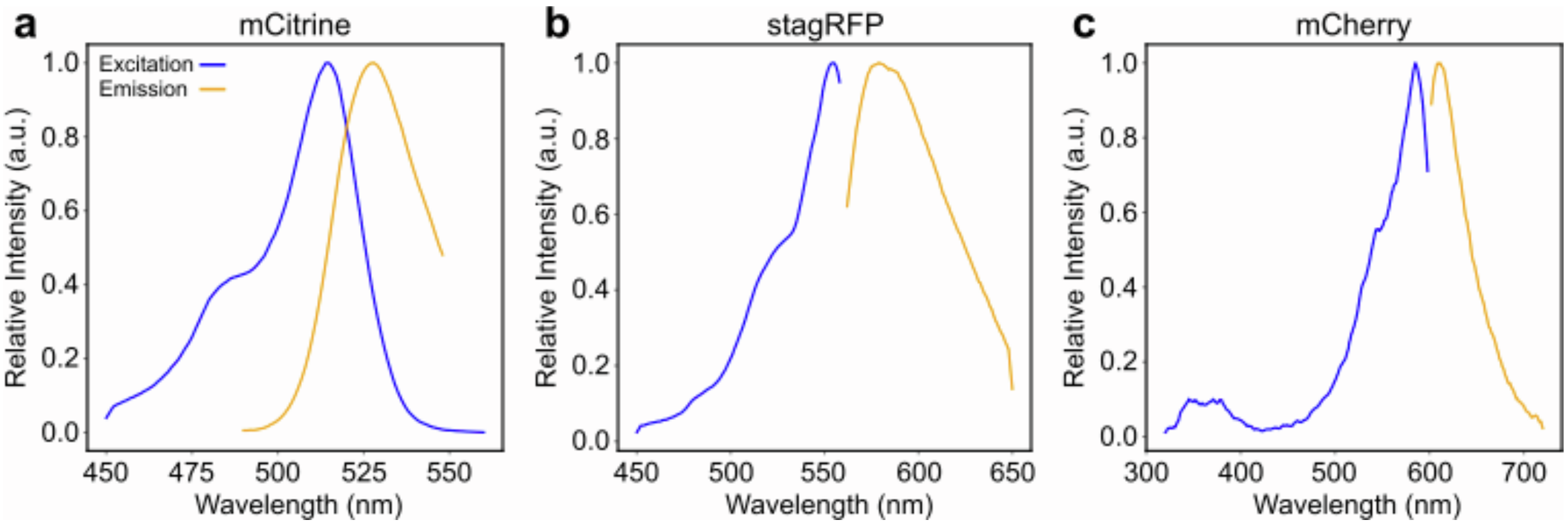

Figure S6: One photon excitation spectra of yellow and red-shifted FPs
Fluorescent proteins expressed in U2OS cells as H2B fusions. Blue curves are the excitation spectra, and the yellow curves are the emission spectra.

a mCitrine

b stagRFP

c mCherry

Spectra were acquired in glass bottom 96 well plates on a fluorescence plate reader. Curves represent averages of multiple repeats.

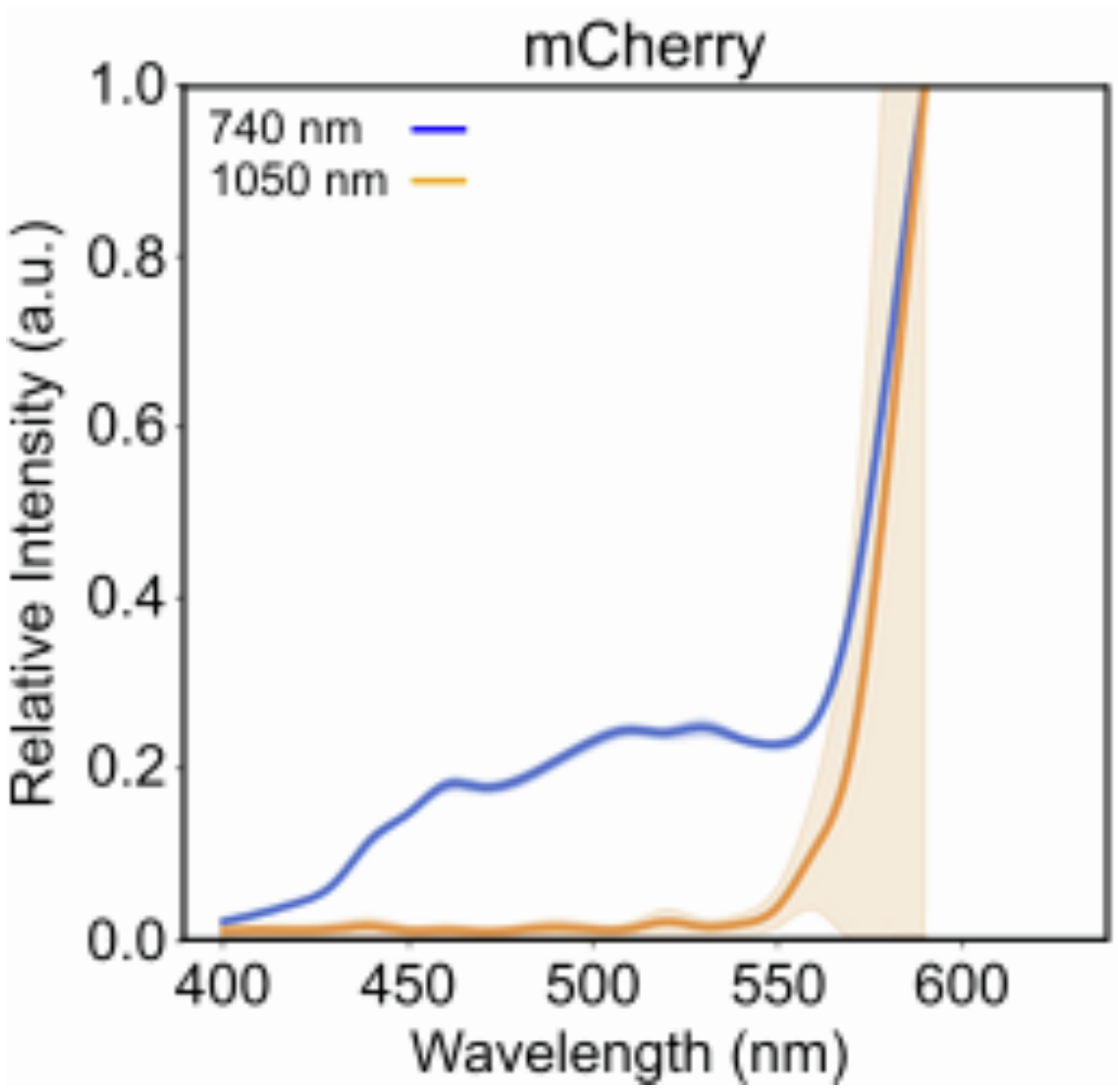

Figure S7: Two-photon emission spectra of mCherry at 740 nm and 1050 nm.
250 nM solution of mCherry in L-15 was excited using 740 nm (blue) and 1050 nm (orange). Values are averaged across an image and normalized to the maximum value. The shaded area represents standard deviation.

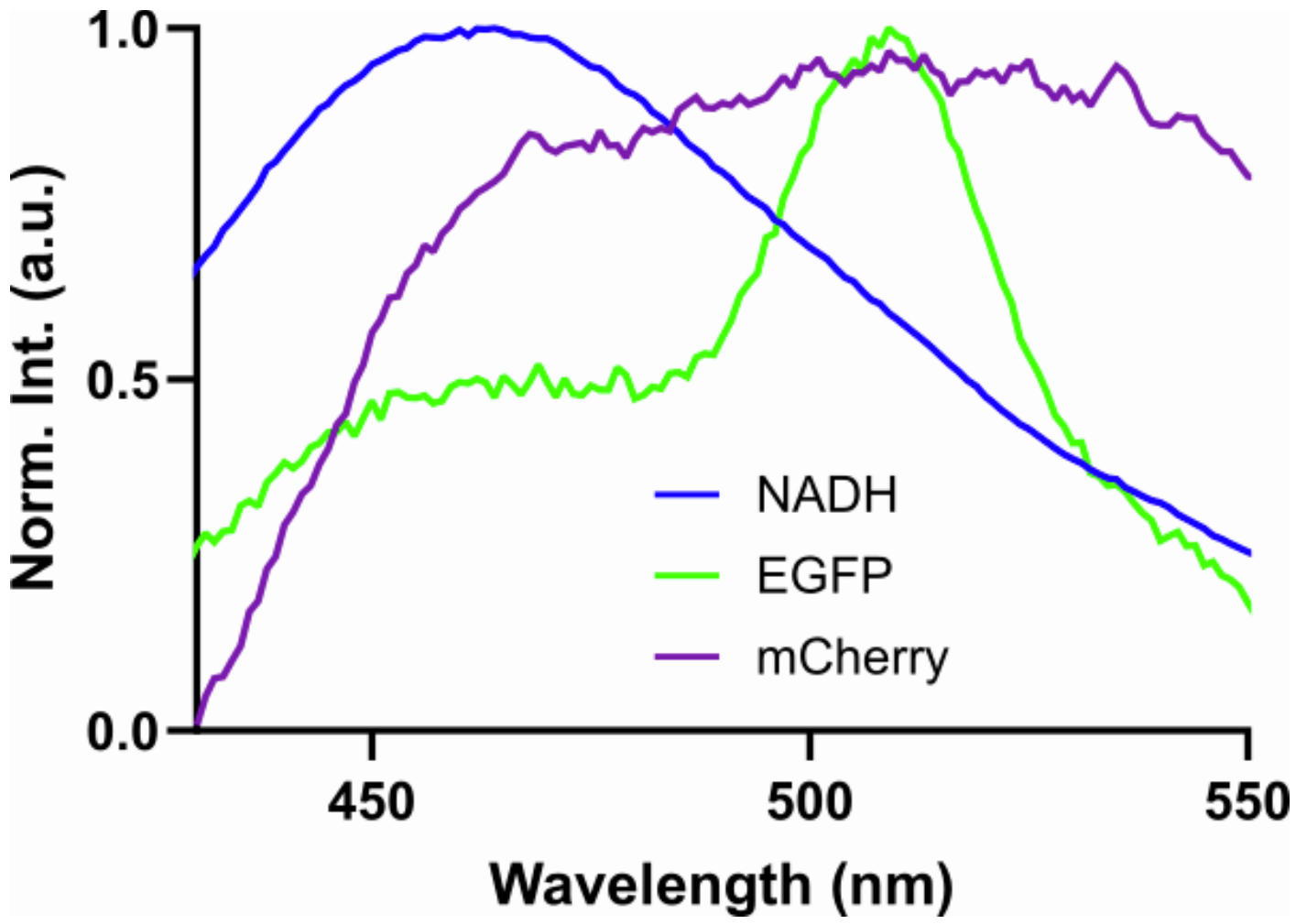

Figure S8: Emission spectra of fluorescent proteins under OPE/UV 370 nm excitation acquired on fluorescence plate reader.
Recombinant proteins, EGFP at 500 nM (green) and mCherry at 1 µM (magenta), and for comparison NADH at 10 mM (blue) were measured in L-15.

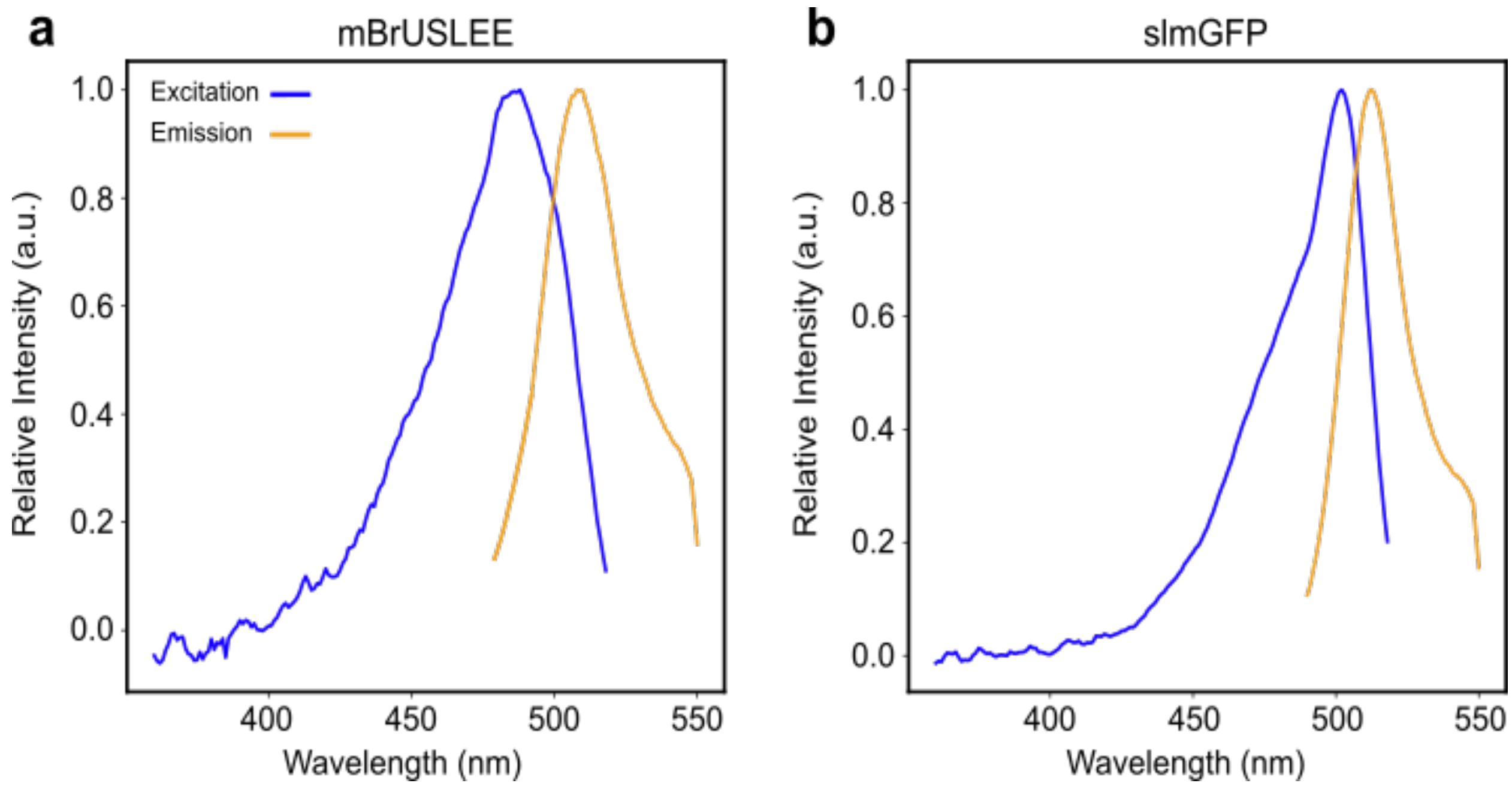

Figure S9: One-photon excitation spectra of short fluorescence lifetime FPs
Fluorescent proteins expressed in U2OS cells as H2B fusions. Blue line is the excitation spectrum, and the yellow line is the emission spectrum.

a mBrUSLEE

b slmGFP

Spectra were acquired in glass bottom 96 well plates on a fluorescence plate reader. Lines represent averages of multiple repeats.

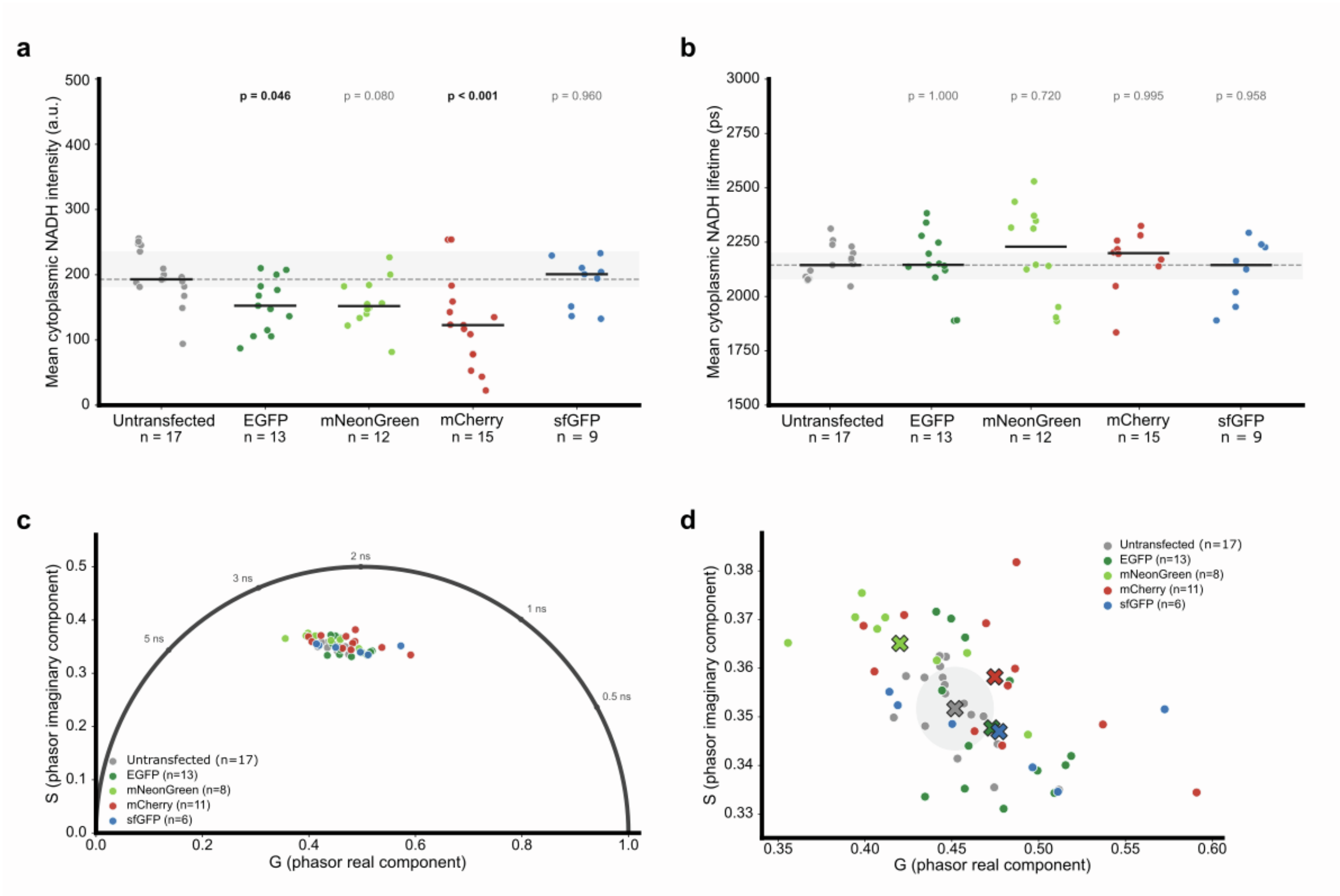

Figure S10: Cytoplasmic NADH measurements in cells expressing nuclear-localized fluorescent proteins.

a Mean cytoplasmic NADH intensity in untransfected cells (n = 17) and cells expressing H2B-tagged EGFP (n = 13), mNeonGreen (n = 12), mCherry (n = 15), or sfGFP (n = 9). Intensity differed among groups (one-way ANOVA, $F(4, 61) = 5.48$, $P < 0.001$).

b Mean cytoplasmic NADH fluorescence lifetime in untransfected cells (n = 17) and cells expressing H2B-tagged EGFP (n = 13), mNeonGreen (n = 12), mCherry (n = 15), or sfGFP (n = 9). No overall difference in lifetime was detected (one-way ANOVA, $F(4, 55) = 0.53$, $P = 0.72$).

In a and b, each circle represents one cell, and short horizontal bars indicate group medians. The dashed line and grey band indicate the median and interquartile range, respectively, of the untransfected control. Dunnett's multiple-comparisons test was used for comparisons with untransfected cells; adjusted p-values are shown above the groups.

c Full phasor distribution for untransfected cells (n = 17) and cells expressing H2B-tagged EGFP (n = 13), mNeonGreen (n = 8), mCherry (n = 11), or sfGFP (n = 6). The solid semicircle denotes the universal semicircle, with the positions of selected single-exponential lifetimes indicated.

d Magnified view of the region occupied by the phasor measurements. Small circles represent individual cells, large X symbols indicate group centroids, and the shaded grey ellipse denotes the +/-1 SD region of the untransfected group. G and S denote the real and imaginary phasor

components, respectively. Intensity is reported in arbitrary units (a.u.) and lifetime in picoseconds (ps).

Table S1: Representative two-photon excitation wavelengths and fluorescence detection windows used for NADH-based metabolic imaging in published studies.

Reference ID	Reference (year)	Sample context	Excitation (nm)	Detection window (nm)	Link
**1**	York 2019	GFP interference	750	460/50	pmc.ncbi.nlm.nih.gov
**2**	Schroeder 2020	Glioblastoma stem cells	740	450/70	pmc.ncbi.nlm.nih.gov
**3**	Baraghis 2011	Cortical tissue	740	460±25	pmc.ncbi.nlm.nih.gov
**4**	Lam 2016	Valve interstitial cells	755	460±20	pmc.ncbi.nlm.nih.gov
**5**	Chen 2021	Brain tissue	780	447/60	thno.org
**6**	Azzarello 2024	Islet α/β cells	740	420–460	pmc.ncbi.nlm.nih.gov
**7**	Sameni 2016	Huntington's disease cells	740	442/46	pmc.ncbi.nlm.nih.gov
**8**	Bragin 2015	Brain microvasculature	740	425–475	pmc.ncbi.nlm.nih.gov
**9**	Ranjit 2019	MEF cells	740	460/40	pmc.ncbi.nlm.nih.gov
**10**	Niederschweiberer 2021	Primary Hippocampal Neurons	730	460/60	frontiersin.org
**11**	Paillon 2024	T lymphocytes	740	460/80	pmc.ncbi.nlm.nih.gov
**12**	Huang 2002	Redox ratio imaging	800/750	410–490	pmc.ncbi.nlm.nih.gov
**13**	Snyder 2023	HIV-infected cells	780	460/55	pmc.ncbi.nlm.nih.gov

Supporting References

1. York EM, Weilinger NL, LeDue JM, MacVicar BA. Green fluorescent protein emission obscures metabolic fluorescent lifetime imaging of NAD(P)H. Biomed Opt Express. 2019;10(9):4381–4394. https://doi.org/10.1364/BOE.10.004381

2. Schroeder AB, Pointer KB, Clark PA, Datta R, Kuo JS, Eliceiri KW. Metabolic mapping of glioblastoma stem cells reveals NADH fluxes associated with glioblastoma phenotype and survival. J Biomed Opt. 2020;25(3):036502. https://doi.org/10.1117/1.JBO.25.3.036502

3. Baraghis E, Devor A, Fang Q, Srinivasan VJ, Wu W, Lesage F, et al. Two-photon microscopy of cortical NADH fluorescence intensity changes: correcting contamination from the hemodynamic response. J Biomed Opt. 2011;16(10):106003. https://doi.org/10.1117/1.3633339

4. Lam NT, Muldoon TJ, Quinn KP, Rajaram N, Balachandran K. Valve interstitial cell contractile strength and metabolic state are dependent on its shape. Integr Biol (Camb). 2016;8(10):1079–1089. https://doi.org/10.1039/c6ib00120c

5. Chen D, Nauen DW, Park HC, Li D, Yuan W, Li A, et al. Label-free imaging of human brain tissue at subcellular resolution for potential rapid intra-operative assessment of glioma surgery. Theranostics. 2021;11(15):7222–7234. https://doi.org/10.7150/thno.59244

6. Azzarello F, Carli F, De Lorenzi V, Tesi M, Marchetti P, Beltram F, et al. Machine-learning-guided recognition of α and β cells from label-free infrared micrographs of living human islets of Langerhans. Sci Rep. 2024;14:14235. https://doi.org/10.1038/s41598-024-65161-7

7. Sameni S, Syed A, Marsh JL, Digman MA. The phasor-FLIM fingerprints reveal shifts from OXPHOS to enhanced glycolysis in Huntington Disease. Sci Rep. 2016;6:34755. https://doi.org/10.1038/srep34755

8. Bragin DE, Statom GL, Hagberg S, Nemoto EM. Increases in microvascular perfusion and tissue oxygenation via pulsed electromagnetic fields in the healthy rat brain. J Neurosurg. 2015;122(5):1239–1247. https://doi.org/10.3171/2014.8.JNS132083

9. Ranjit S, Malacrida L, Stakic M, Gratton E. Determination of the metabolic index using the fluorescence lifetime of free and bound nicotinamide adenine dinucleotide using the phasor approach. J Biophotonics. 2019;12(11):e201900156. https://doi.org/10.1002/jbio.201900156

10. Niederschweiberer MA, Schaefer PM, Singh LN, Lausser L, Bhosale D, Hesse R, et al. NADH fluorescence lifetime imaging microscopy reveals selective mitochondrial dysfunction in neurons overexpressing Alzheimer's disease-related proteins. Front Mol Biosci. 2021;8:671274. https://doi.org/10.3389/fmolb.2021.671274

11. Paillon N, Ung TPL, Dogniaux S, Stringari C, Hivroz C. Label-free single-cell live imaging reveals fast metabolic switch in T lymphocytes. Mol Biol Cell. 2024;35(1):ar11. https://doi.org/10.1091/mbc.E23-01-0009

12. Huang S, Heikal AA, Webb WW. Two-photon fluorescence spectroscopy and microscopy of NAD(P)H and flavoprotein. Biophys J. 2002;82(5):2811–2825. https://doi.org/10.1016/S0006-3495(02)75621-X

13. Snyder GA, Kumar S, Lewis GK, Ray K. Two-photon fluorescence lifetime imaging microscopy of NADH metabolism in HIV-1 infected cells and tissues. Front Immunol. 2023;14:1213180. https://doi.org/10.3389/fimmu.2023.1213180
